# Supplementary material for: Spectral efficiency and BER analysis of RNN based hybrid precoding for cell free massive MIMO under terahertz communication
Source: PLoS One. 2025 Aug 14;20(8):e0328499. doi: 10.1371/journal.pone.0328499 (PMC12352664; doi:10.1371/journal.pone.0328499)
Supplement: S2 Code — Script used for generating the results presented in the manuscript. (DOCX) [file pone.0328499.s002.docx]

SPECTRAL EFFICIENCY AND BER ANALYSIS OF HYBRID PRECODING FOR CELL FREE MASSIVE MIMO UNDER THz COMMUNICATION USING RECURRENT NEURAL NETWORKS

Machine Learning Training Model Code LSTM (Advanced RNN)

# LSTM (advanced RNN) based ML Training Model

import numpy as np

import pandas as pd

import matplotlib.pyplot as plt

from sklearn.model_selection import train_test_split

from sklearn.preprocessing import MinMaxScaler

from tensorflow.keras.models import Sequential

from tensorflow.keras.layers import LSTM, Dense, Dropout

from tensorflow.keras.callbacks import ReduceLROnPlateau, Callback

from tensorflow.keras.optimizers import Adam

from sklearn.metrics import mean_squared_error, mean_absolute_error, r2_score

from tensorflow.keras import backend as K

# Custom callback for logging learning rates

class LearningRateLogger(Callback):

    def on_epoch_end(self, epoch, logs=None):

        logs = logs or {}

        logs['learning_rate'] = K.get_value(self.model.optimizer.lr)

# Load the dataset (j. xlxs generated by dataset generating code and stored in the path as shown below)

data = pd.read_excel('/content/drive/MyDrive/Dataset.xlsx')

# Select relevant columns for prediction (dataset parametrs)

X = data[['channel_gains_real', 'channel_gains_imag',

          'channel_phases_real', 'channel_phases_imag',

          'spatial_correlation', 'noise_power', 'transmitted_signal',

          'transmitted_power', 'path_loss', 'shadowing', 'fading', 'user_speed', 'V_B_real', 'V_B_imag', 'V_R_real', 'V_R_imag', 'w_R_real', 'w_R_imag']]

y = data[['V_B_real', 'V_B_imag', 'V_R_real', 'V_R_imag', 'w_R_real', 'w_R_imag']]

# Preprocess the data

scaler_X = MinMaxScaler()

X_scaled = scaler_X.fit_transform(X)

scaler_y = MinMaxScaler()

y_scaled = scaler_y.fit_transform(y)

# Split the data into training, validation, and test sets

X_train, X_temp, y_train, y_temp = train_test_split(X_scaled, y_scaled, test_size=0.3, random_state=42)

X_val, X_test, y_val, y_test = train_test_split(X_temp, y_temp, test_size=0.5, random_state=42)

# Reshape the data for LSTM input

X_train = X_train.reshape(X_train.shape[0], 1, X_train.shape[1])

X_val = X_val.reshape(X_val.shape[0], 1, X_val.shape[1])

X_test = X_test.reshape(X_test.shape[0], 1, X_test.shape[1])

# Define ReduceLROnPlateau callback

reduce_lr = ReduceLROnPlateau(monitor='val_loss', factor=0.2, patience=10, min_lr=1e-6)

# Instantiate the LearningRateLogger

lr_logger = LearningRateLogger()

# Define the LSTM model (Advance RNN)

model = Sequential()

model.add(LSTM(128, input_shape=(X_train.shape[1], X_train.shape[2]), return_sequences=True))

model.add(Dropout(0.5))

model.add(LSTM(64, return_sequences=False))

model.add(Dropout(0.5))

model.add(Dense(6, activation='tanh'))

# Compile the model with a learning rate of 0.0001

model.compile(optimizer=Adam(lr=0.0001), loss='mse', metrics=['accuracy'])

# Train the model

history = model.fit(X_train, y_train, epochs=250, batch_size=256,

                    validation_data=(X_val, y_val), callbacks=[reduce_lr, lr_logger], verbose=1)

# Evaluate the model

y_pred = model.predict(X_test)

# Convert scaled predictions back to original scale

y_pred_inv = scaler_y.inverse_transform(y_pred)

y_test_inv = scaler_y.inverse_transform(y_test)

# Calculate performance metrics

mse = mean_squared_error(y_test_inv, y_pred_inv)

mae = mean_absolute_error(y_test_inv, y_pred_inv)

r2 = r2_score(y_test_inv, y_pred_inv)

print(f"Mean Squared Error (MSE): {mse}")

print(f"Mean Absolute Error (MAE): {mae}")

print(f"R^2 Score: {r2}")

# Plot training & validation loss values

plt.figure(figsize=(12, 6))

plt.plot(history.history['loss'], label='Training Loss', color='blue')

plt.plot(history.history['val_loss'], label='Validation Loss', color='red')

plt.title('Training and Validation Loss')

plt.xlabel('Epochs')

plt.ylabel('Loss')

plt.legend()

plt.show()

# Plot training & validation accuracy values

plt.figure(figsize=(12, 6))

plt.plot(history.history['accuracy'], label='Training Accuracy', color='green')

plt.plot(history.history['val_accuracy'], label='Validation Accuracy', color='orange')

plt.title('Training and Validation Accuracy')

plt.xlabel('Epochs')

plt.ylabel('Accuracy')

plt.legend()

plt.show()

# Plot learning rate vs. epoch

plt.figure(figsize=(12, 6))

plt.plot(history.history['learning_rate'], label='Learning Rate', color='purple')

plt.title('Learning Rate over Epochs')

plt.xlabel('Epochs')

plt.ylabel('Learning Rate')

plt.legend()

plt.show()

Loading ML Trained Model, Making Prediction and Saving it to Excel

# Save the trained model

model.save('trained_model.h5')

# Load the trained model

loaded_model = tf.keras.models.load_model('trained_model.h5')

# Make predictions

y_pred = loaded_model.predict(X_test)

# Convert scaled predictions back to original scale

y_pred_inv = scaler_y.inverse_transform(y_pred)

# Save the predictions to an Excel file

df = pd.DataFrame(y_pred_inv, columns=['V_B_real', 'V_B_imag', 'V_R_real', 'V_R_imag', 'w_R_real', 'w_R_imag'])

df.to_excel('predictions.xlsx', index=False)

Spectral Efficiency vs SNR

import pandas as pd

import numpy as np

import matplotlib.pyplot as plt

# Load the predicted results from the machine learning modelthat is saved in excel form

predicted_df = pd.read_excel('/content/drive/MyDrive/predictions_with_names.xlsx')

#  the predicted results contain columns 'V_B_real', 'V_B_imag', 'V_R_real', 'V_R_imag', 'w_R_real', 'w_R_imag' (digital precoder Vb, analog precoder Vr and analog combiner Wr)

V_B_real = predicted_df['V_B_real'].values

V_B_imag = predicted_df['V_B_imag'].values

V_R_real = predicted_df['V_R_real'].values

V_R_imag = predicted_df['V_R_imag'].values

w_R_real = predicted_df['w_R_real'].values

w_R_imag = predicted_df['w_R_imag'].values

# Given parameters

N_T = 256 #number of transmitting antennas

N_R = 64 #number of receiving antennas

N_P = 5

N_C = 10

frequency = 1e12  # 1 THz in Hz

sigma2 = 10**(-10)  # Noise variance

K = 5

P = 10**(40 / 10)  # Transmit power in linear scale

sigma2 = 1

p = 10

B = 20e9  # Bandwidth in GHz (assuming 20 GHz)

# Generate random angles for clusters and paths

phi_r = np.random.uniform(0, 2 * np.pi, (N_C, N_P))

phi_t = np.random.uniform(0, 2 * np.pi, (N_C, N_P))

# Generate complex Gaussian channel gains

channel_gains = (np.random.randn(N_C, N_P) + 1j * np.random.randn(N_C, N_P)) / np.sqrt(2)

# Define antenna array response vectors

def array_response(angle, N):

    return (1 / np.sqrt(N)) * np.exp(1j * np.pi * np.arange(N) * np.sin(angle))

# RNN based hybrid Precoder

# Calculate the channel matrix H_k for each user

H_k = []

for k in range(K):

    H_k_user = np.zeros((N_R, N_T), dtype=complex)

    for i in range(N_C):

        for j in range(N_P):

            ar = array_response(phi_r[i, j], N_R)

            at = array_response(phi_t[i, j], N_T)

            H_k_user += channel_gains[i, j] * np.outer(ar, at)

    H_k_user *= np.sqrt((N_T * N_R) / (N_C * N_P))

    H_k.append(H_k_user)

# Use only the necessary elements from the predicted arrays

W_R = (w_R_real[:N_R * N_C] + 1j * w_R_imag[:N_R * N_C]).reshape(N_C, N_R).T

V_R = (V_R_real[:N_T * N_C] + 1j * V_R_imag[:N_T * N_C]).reshape(N_C, N_T).T

# Calculate the effective channel h_(l_k)

h_lk = []

for k in range(K):

    H_k_user = H_k[k]

    h_lk_user = np.conjugate(W_R).T @ H_k_user @ V_R

    h_lk.append(h_lk_user)

# Compute SINR for each user terminal for the RNN based hybrid precoder

SINR = []

for k in range(K):

    numerator = (P / K) * np.abs(np.dot(h_lk[k], V_B_real[k] + 1j * V_B_imag[k]))**2

    denominator = np.sum([(P / K) * np.abs(np.dot(h_lk[j], V_B_real[j] + 1j * V_B_imag[j]))**2 for j in range(K) if j != k]) + sigma2

    SINR_val = numerator / denominator

    SINR.append(SINR_val)

# Convert SINR to a numpy array

SINR = np.array(SINR)

# Convert SINR from linear scale to dB scale

SINR_dB = 10 * np.log10(SINR)

# Calculate sumrate

sumrate = np.log2(1 + SINR)

# Calculate spectral efficiency by dividing sumrate by bandwidth

spectral_efficiency = sumrate / bandwidth

# MMSE PRECODER

# Initialize H_k matrix for MMSE precoder

H_k = np.zeros((K, N_R, N_T), dtype=complex)

# Compute H_k

for k in range(K):

    for i in range(N_C):

        for l in range(N_P):

            a_r = array_response_vector(phi_r[i, l], N_R)

            a_t = array_response_vector(phi_t[i, l], N_T)

            H_k[k] += np.sqrt((N_T * N_R) / (N_C * N_P)) * a_ik_il[i, l] * np.outer(a_r, a_t)

# Compute A_k, analog precoder v_R_k, B_K, G_k, and analog combiner w_R_k

A_k = np.zeros((K, N_T, N_R), dtype=complex)

v_R_k = np.zeros((K, N_T), dtype=complex)

B_K = np.zeros((K, N_R), dtype=complex)

G_k = np.zeros((K, N_R, N_R), dtype=complex)

w_R_k = np.zeros((K, N_R), dtype=complex)

for k in range(K):

    A_k[k] = np.linalg.inv(H_k[k].conj().T @ H_k[k] + (K * sigma2 / P) * np.eye(N_T)) @ H_k[k].conj().T

    A_k_phase_angles = np.angle(A_k[k]).flatten()

    v_R_k[k] = 1 / np.sqrt(N_T) * np.exp(1j * A_k_phase_angles[:N_T])

    B_K[k] = (H_k[k] @ v_R_k[k][:, np.newaxis]).flatten()

    G_k[k] = np.linalg.inv(B_K[k].conj().T @ B_K[k] + (K * sigma2 / P) * np.eye(N_R)) @ B_K[k].conj().T @ B_K[k]

    G_k_phase_angles = np.angle(G_k[k]).flatten()

    w_R_k[k] = 1 / np.sqrt(N_R) * np.exp(1j * G_k_phase_angles[:N_R])

# Compute the effective channel h_e_k

h_e_k = np.zeros((K, N_R), dtype=complex)

for k in range(K):

    h_e_k[k] = w_R_k[k].conj().T @ H_k[k] @ v_R_k[k]

# Compute the digital precoder V_B

H_e = np.array(h_e_k)

V_B = np.linalg.inv(H_e.conj().T @ H_e + (K * sigma2 / P) * np.eye(K)) @ H_e.conj().T

# Compute SINR in dB for each user terminal

SINR_dB = []

for k in range(K):

    numerator = (P / K) * np.abs(h_e_k[k] @ V_B[:, k])**2

    denominator = np.sum([(P / K) * np.abs(h_e_k[k] @ V_B[:, j])**2 for j in range(K) if j != k]) + sigma2

    SINR_k = numerator / denominator

    SINR_dB.append(10 * np.log10(SINR_k))

# Compute sum-rate for each user terminal

sum_rate = sum([np.log2(1 + 10**(SINR / 10)) for SINR in SINR_dB])

# Compute spectral efficiency per Hertz

spectral_efficiency_per_Hz = sum_rate / B

#zeroforcing

# Initialize H_k matrix for zero forcing

H_k = np.zeros((N_C, N_P), dtype=complex)

# Compute H_k

for i in range(N_C):

    for j in range(N_P):

        cos_term = np.cos(phi_r[i] - phi_t[j])

        H_k[i, j] = np.sqrt((N_T * N_R) / (N_C * N_P)) * channel_gains[i, j] * cos_term

# Zero Forcing Precoder

V_ZF = np.linalg.pinv(H_k)

# Normalize precoder

V_ZF = V_ZF / np.linalg.norm(V_ZF, axis=0)

# Effective channel

H_e = H_k @ V_ZF

# Compute SINR for each user terminal

SINR = []

for i in range(N_C):

    h_l_k = H_e[i, :]

    numerator = (p / sigma2) * np.abs(h_l_k)**2

    interference = np.sum([(p / sigma2) * np.abs(H_e[j, :])**2 for j in range(N_C) if j != i])

    denominator = interference + sigma2

    SINR_val = numerator / denominator

    SINR.append(SINR_val)

SINR = np.array(SINR)

# Compute achievable data rate (r_k) for each user terminal

r_k = np.log2(1 + SINR)

# Compute spectral efficiency (SE_k) for each user terminal

SE_k = r_k / B

# Compute sum-rate

sum_rate = np.sum(r_k)

# MRT Precoder

# Compute H_k

for i in range(N_C):

    for j in range(N_P):

        cos_term = np.cos(phi_r[i] - phi_t[j])

        H_k[i, j] = np.sqrt((N_T * N_R) / (N_C * N_P)) * channel_gains[i, j] * cos_term

# Maximum Ratio Transmission Precoder

V_MRT = H_k.conj().T

# Normalize precoder

V_MRT = V_MRT / np.linalg.norm(V_MRT, axis=0)

# Effective channel

H_e = H_k @ V_MRT

# Compute SINR for each user terminal

SINR = []

for i in range(N_C):

    h_l_k = H_e[i, :]

    numerator = (p / sigma2) * np.abs(h_l_k)**2

    interference = np.sum([(p / sigma2) * np.abs(H_e[j, :])**2 for j in range(N_C) if j != i])

    denominator = interference + sigma2

    SINR_val = numerator / denominator

    SINR.append(SINR_val)

SINR = np.array(SINR)

# Compute achievable data rate (r_k) for each user terminal

r_k = np.log2(1 + SINR)

# Compute spectral efficiency (SE_k) for each user terminal

SE_k = r_k / B

# Compute sum-rate

sum_rate = np.sum(r_k)

# Alamouti

# Initialize H_k matrix

H_k = np.zeros((N_C, N_P, N_T), dtype=complex)

# Compute H_k

for i in range(N_C):

    for j in range(N_P):

        cos_term = np.cos(phi_r[i] - phi_t[j])

        for k in range(N_T):

            H_k[i, j, k] = np.sqrt((N_T * N_R) / (N_C * N_P)) * channel_gains[i, j] * cos_term

# Alamouti precoding

V_Alamouti = np.zeros((N_T, 2), dtype=complex)

V_Alamouti[0, 0] = 1

V_Alamouti[1, 1] = 1

V_Alamouti[0, 1] = -1

V_Alamouti[1, 0] = 1

# Effective channel for Alamouti

H_e = np.zeros((N_C, 2), dtype=complex)

for i in range(N_C):

    H_e[i, :] = np.dot(H_k[i, :, :], V_Alamouti[:, 0]) + np.dot(H_k[i, :, :], V_Alamouti[:, 1])

# Compute SINR for each user terminal

SINR = []

for i in range(N_C):

    h_l_k = H_e[i, :]

    numerator = (p / sigma2) * np.abs(h_l_k)**2

    interference = np.sum([(p / sigma2) * np.abs(H_e[j, :])**2 for j in range(N_C) if j != i])

    denominator = interference + sigma2

    SINR_val = numerator / denominator

    SINR.append(SINR_val)

SINR = np.array(SINR)

# Compute achievable data rate (r_k) for each user terminal

r_k = np.log2(1 + SINR)

# Compute spectral efficiency (SE_k) for each user terminal

SE_k = r_k / B

# Compute sum-rate

sum_rate = np.sum(r_k)

# Plot Spectral Efficiency vs SNR

fig, ax = plt.subplots(figsize=(10, 6))

ax.plot(snr_db, se_mmse_vals, label='MMSE', marker='o', markersize=8, markevery=3)

ax.plot(snr_db, se_zf_vals, label='ZF', marker='x', markersize=8, markevery=3)

ax.plot(snr_db, se_mrt_vals, label='MRT', marker='s', markersize=8, markevery=3)

ax.plot(snr_db, se_alamouti_vals, label='Alamouti', marker='d', markersize=8, markevery=3)

ax.plot(snr_db, se_ml_vals, label='RNN-based', marker='^', markersize=8, markevery=3)

ax.set_xlabel('SNR (dB)')

ax.set_ylabel('Spectral Efficiency (bps/Hz)')

ax.set_title('Spectral Efficiency vs SNR')

ax.legend(labelspacing=1, loc='upper left')

ax.grid(True, linestyle='--', linewidth=0.5)

ax.set_ylim(0, 10)

ax.set_xlim(-20, 30)

# Add zoomed inset inside the main plot

ax_inset = inset_axes(ax, width="50%", height="50%", loc="upper left",

                      bbox_to_anchor=(0.35, 0.35, 0.5, 0.5), bbox_transform=ax.transAxes)

ax_inset.plot(snr_db, se_mmse_vals, label='MMSE', marker='o', markersize=8, markevery=3)

ax_inset.plot(snr_db, se_zf_vals, label='ZF', marker='x', markersize=8, markevery=3)

ax_inset.plot(snr_db, se_mrt_vals, label='MRT', marker='s', markersize=8, markevery=3)

ax_inset.plot(snr_db, se_alamouti_vals, label='Alamouti', marker='d', markersize=8, markevery=3)

ax_inset.plot(snr_db, se_ml_vals, label='RNN-based', marker='^', markersize=8, markevery=3)

ax_inset.grid(True, linestyle='--', linewidth=0.5)

# Remove the ticks and labels from the inset plot

ax_inset.tick_params(left=False, bottom=False, labelleft=False, labelbottom=False)

# Add a rectangle to highlight the zoomed area

rect = plt.Rectangle((10, 3), 5, 3, linewidth=1, edgecolor='black', facecolor='none', linestyle='--')

ax.add_patch(rect)

plt.show()

Spectral Efficiency v_s_ Number of Users

import numpy as np

import pandas as pd

import matplotlib.pyplot as plt

# Load the predicted results from the machine learning modelthat is saved in excel form

predicted_df = pd.read_excel('/content/drive/MyDrive/predictions_with_names.xlsx')

# Extract the predicted results (digital precoder Vb, analog precoder Vr and analog combiner Wr)

V_B_real = predicted_df['V_B_real'].values

V_B_imag = predicted_df['V_B_imag'].values

V_R_real = predicted_df['V_R_real'].values

V_R_imag = predicted_df['V_R_imag'].values

w_R_real = predicted_df['w_R_real'].values

w_R_imag = predicted_df['w_R_imag'].values

# Given parameters

N_T = 256 #number of transmitter antenna

N_R = 64 #number of receiver antenna

N_P = 5

N_C = 10

frequency = 1e12  # 1 THz in Hz

sigma2 = 10**(-10)  # Noise variance

P = 10**(40 / 10)  # Transmit power in linear scale

B = 20e9  # Bandwidth in Hz (assuming 20 GHz)

# Generate random angles for clusters and paths

phi_r = np.random.uniform(0, 2 * np.pi, (N_C, N_P))

phi_t = np.random.uniform(0, 2 * np.pi, (N_C, N_P))

# Generate complex Gaussian channel gains

channel_gains = (np.random.randn(N_C, N_P) + 1j * np.random.randn(N_C, N_P)) / np.sqrt(2)

# Define antenna array response vectors

def array_response(angle, N):

    return (1 / np.sqrt(N)) * np.exp(1j * np.pi * np.arange(N) * np.sin(angle))

# Initialize lists to store results

se_mmse_vals = []

se_zf_vals = []

se_mrt_vals = []

se_alamouti_vals = []

se_ml_vals = []

# Loop over different numbers of users

num_users = np.arange(2, 51)

for K in num_users:

    # Calculate the channel matrix H_k for each user

    H_k = []

    for k in range(K):

        H_k_user = np.zeros((N_R, N_T), dtype=complex)

        for i in range(N_C):

            for j in range(N_P):

                ar = array_response(phi_r[i, j], N_R)

                at = array_response(phi_t[i, j], N_T)

                H_k_user += channel_gains[i, j] * np.outer(ar, at)

        H_k_user *= np.sqrt((N_T * N_R) / (N_C * N_P))

        H_k.append(H_k_user)

    # Use only the necessary elements from the predicted arrays

    W_R = (w_R_real[:N_R * K] + 1j * w_R_imag[:N_R * K]).reshape(K, N_R).T

    V_R = (V_R_real[:N_T * K] + 1j * V_R_imag[:N_T * K]).reshape(K, N_T).T

    # Calculate the effective channel h_(l_k)

    h_lk = []

    for k in range(K):

        H_k_user = H_k[k]

        h_lk_user = np.conjugate(W_R).T @ H_k_user @ V_R

        h_lk.append(h_lk_user)

    # Compute SINR for each user terminal for the original method

    SINR = []

    for k in range(K):

        numerator = (P / K) * np.abs(np.dot(h_lk[k], V_B_real[k] + 1j * V_B_imag[k]))**2

        denominator = np.sum([(P / K) * np.abs(np.dot(h_lk[j], V_B_real[j] + 1j * V_B_imag[j]))**2 for j in range(K) if j != k]) + sigma2

        SINR_val = numerator / denominator

        SINR.append(SINR_val)

    # Convert SINR to a numpy array

    SINR = np.array(SINR)

    # Convert SINR from linear scale to dB scale

    SINR_dB = 10 * np.log10(SINR)

    # Calculate sumrate

    sumrate = np.sum(np.log2(1 + SINR))

    # Calculate spectral efficiency by dividing sumrate by bandwidth

    se_ml = sumrate / B

    se_ml_vals.append(se_ml)

    # MMSE PRECODER

    # Initialize H_k matrix for MMSE precoder

    H_k_mmse = np.zeros((K, N_R, N_T), dtype=complex)

    # Compute H_k

    for k in range(K):

        for i in range(N_C):

            for l in range(N_P):

                ar = array_response(phi_r[i, l], N_R)

                at = array_response(phi_t[i, l], N_T)

                H_k_mmse[k] += np.sqrt((N_T * N_R) / (N_C * N_P)) * channel_gains[i, l] * np.outer(ar, at)

    # Compute A_k, analog precoder v_R_k, B_K, G_k, and analog combiner w_R_k

    A_k = np.zeros((K, N_T, N_R), dtype=complex)

    v_R_k = np.zeros((K, N_T), dtype=complex)

    B_K = np.zeros((K, N_R), dtype=complex)

    G_k = np.zeros((K, N_R, N_R), dtype=complex)

    w_R_k = np.zeros((K, N_R), dtype=complex)

    for k in range(K):

        A_k[k] = np.linalg.inv(H_k_mmse[k].conj().T @ H_k_mmse[k] + (K * sigma2 / P) * np.eye(N_T)) @ H_k_mmse[k].conj().T

        A_k_phase_angles = np.angle(A_k[k]).flatten()

        v_R_k[k] = 1 / np.sqrt(N_T) * np.exp(1j * A_k_phase_angles[:N_T])

        B_K[k] = (H_k_mmse[k] @ v_R_k[k][:, np.newaxis]).flatten()

        G_k[k] = np.linalg.inv(B_K[k].conj().T @ B_K[k] + (K * sigma2 / P) * np.eye(N_R)) @ B_K[k].conj().T @ B_K[k]

        G_k_phase_angles = np.angle(G_k[k]).flatten()

        w_R_k[k] = 1 / np.sqrt(N_R) * np.exp(1j * G_k_phase_angles[:N_R])

    # Compute the effective channel h_e_k

    h_e_k = np.zeros((K, N_R), dtype=complex)

    for k in range(K):

        h_e_k[k] = w_R_k[k].conj().T @ H_k_mmse[k] @ v_R_k[k]

    # Compute the digital precoder V_B

    H_e = np.array(h_e_k)

    V_B_mmse = np.linalg.inv(H_e.conj().T @ H_e + (K * sigma2 / P) * np.eye(K)) @ H_e.conj().T

    # Compute SINR in dB for each user terminal

    SINR_dB_mmse = []

    for k in range(K):

        numerator = (P / K) * np.abs(h_e_k[k] @ V_B_mmse[:, k])**2

        denominator = np.sum([(P / K) * np.abs(h_e_k[k] @ V_B_mmse[:, j])**2 for j in range(K) if j != k]) + sigma2

        SINR_k = numerator / denominator

        SINR_dB_mmse.append(10 * np.log10(SINR_k))

    # Compute sum-rate for each user terminal

    sum_rate_mmse = np.sum([np.log2(1 + 10**(SINR / 10)) for SINR in SINR_dB_mmse])

    # Compute spectral efficiency per Hertz

    spectral_efficiency_mmse = sum_rate_mmse / B

    se_mmse_vals.append(spectral_efficiency_mmse)

    # Zero Forcing Precoder

    V_ZF = np.linalg.pinv(H_e)

    # Normalize precoder

    V_ZF = V_ZF / np.linalg.norm(V_ZF, axis=0)

    # Effective channel

    H_e_zf = H_e @ V_ZF

    # Compute SINR for each user terminal

    SINR_zf = []

    for k in range(K):

        h_l_k = H_e_zf[k, :]

        numerator = (P / K) * np.abs(h_l_k)**2

        interference = np.sum([(P / K) * np.abs(H_e_zf[j, :])**2 for j in range(K) if j != k])

        denominator = interference + sigma2

        SINR_val = numerator / denominator

        SINR_zf.append(SINR_val)

    SINR_zf = np.array(SINR_zf)

    # Compute achievable data rate (r_k) for each user terminal

    r_k_zf = np.log2(1 + SINR_zf)

    # Compute spectral efficiency (SE_k) for each user terminal

    SE_k_zf = r_k_zf / B

    # Compute sum-rate for ZF

    sum_rate_zf = np.sum(r_k_zf)

    se_zf_vals.append(sum_rate_zf)

    # MRT Precoder

    V_MRT = H_e.conj().T

    # Normalize precoder

    V_MRT = V_MRT / np.linalg.norm(V_MRT, axis=0)

    # Effective channel

    H_e_mrt = H_e @ V_MRT

    # Compute SINR for each user terminal

    SINR_mrt = []

    for k in range(K):

        h_l_k = H_e_mrt[k, :]

        numerator = (P / K) * np.abs(h_l_k)**2

        interference = np.sum([(P / K) * np.abs(H_e_mrt[j, :])**2 for j in range(K) if j != k])

        denominator = interference + sigma2

        SINR_val = numerator / denominator

        SINR_mrt.append(SINR_val)

    SINR_mrt = np.array(SINR_mrt)

    # Compute achievable data rate (r_k) for each user terminal

    r_k_mrt = np.log2(1 + SINR_mrt)

    # Compute spectral efficiency (SE_k) for each user terminal

    SE_k_mrt = r_k_mrt / B

    # Compute sum-rate for MRT

    sum_rate_mrt = np.sum(r_k_mrt)

    se_mrt_vals.append(sum_rate_mrt)

    # Alamouti Precoding

    if N_T < 2:

        print("Alamouti requires at least 2 transmit antennas.")

        break

    # Initialize H_k matrix

    H_k_alamouti = np.zeros((K, N_R, N_T), dtype=complex)

    # Compute H_k

    for k in range(K):

        for i in range(N_C):

            for j in range(N_P):

                ar = array_response(phi_r[i, j], N_R)

                at = array_response(phi_t[i, j], N_T)

                H_k_alamouti[k] += channel_gains[i, j] * np.outer(ar, at)

        H_k_alamouti[k] *= np.sqrt((N_T * N_R) / (N_C * N_P))

    # Alamouti precoding

    V_Alamouti = np.zeros((N_T, 2), dtype=complex)

    V_Alamouti[0, 0] = 1

    V_Alamouti[1, 1] = 1

    V_Alamouti[0, 1] = -1

    V_Alamouti[1, 0] = 1

    # Effective channel for Alamouti

    H_e_alamouti = np.zeros((K, 2), dtype=complex)

    for k in range(K):

        H_e_alamouti[k, :] = np.dot(H_k_alamouti[k, :, :], V_Alamouti[:, 0]) + np.dot(H_k_alamouti[k, :, :], V_Alamouti[:, 1])

    # Compute SINR for each user terminal

    SINR_alamouti = []

    for k in range(K):

        h_l_k = H_e_alamouti[k, :]

        numerator = (P / K) * np.abs(h_l_k)**2

        interference = np.sum([(P / K) * np.abs(H_e_alamouti[j, :])**2 for j in range(K) if j != k])

        denominator = interference + sigma2

        SINR_val = numerator / denominator

        SINR_alamouti.append(SINR_val)

    SINR_alamouti = np.array(SINR_alamouti)

    # Compute achievable data rate (r_k) for each user terminal

    r_k_alamouti = np.log2(1 + SINR_alamouti)

    # Compute spectral efficiency (SE_k) for each user terminal

    SE_k_alamouti = r_k_alamouti / B

    # Compute sum-rate for Alamouti

    sum_rate_alamouti = np.sum(r_k_alamouti)

    se_alamouti_vals.append(sum_rate_alamouti)

# Plot Spectral Efficiency vs Number of Users

plt.figure(figsize=(10, 6))

plt.plot(num_users, se_mmse_vals, marker='o', label='MMSE', markevery=5)

plt.plot(num_users, se_zf_vals, marker='s', label='ZF', markevery=5)  # Square marker

plt.plot(num_users, se_mrt_vals, marker='^', label='MRT', markevery=5)  # Triangle marker

plt.plot(num_users, se_alamouti_vals, marker='d', label='Alamouti', markevery=5)  # Diamond marker

plt.plot(num_users, se_ml_vals, marker='x', label='RNN-based', markevery=5)  # X marker

plt.xlabel('Number of Users')

plt.ylabel('Spectral Efficiency (bps/Hz)')

plt.title('Spectral Efficiency vs Number of Users')

plt.legend()

plt.grid(True, linestyle='--', linewidth=1)

plt.show()

Spectral Efficiency vs Number of RF Chains

import numpy as np

import pandas as pd

import matplotlib.pyplot as plt

# Load the predicted results from the Excel file

predicted_df = pd.read_excel('/content/drive/MyDrive/predictions_with_names.xlsx')

# Extract the predicted results (digital precoder Vb, analog precoder Vr and analog combiner Wr)

V_B_real = predicted_df['V_B_real'].values

V_B_imag = predicted_df['V_B_imag'].values

V_R_real = predicted_df['V_R_real'].values

V_R_imag = predicted_df['V_R_imag'].values

w_R_real = predicted_df['w_R_real'].values

w_R_imag = predicted_df['w_R_imag'].values

# Given parameters

N_T = 256

N_R = 64

N_P = 5

N_C = 10

frequency = 1e12  # 1 THz in Hz

sigma2 = 10**(-10)  # Noise variance

Rf= [4, 5, 6, 7, 8]  #rf chain

P = 10**(40 / 10)  # Transmit power in linear scale

B = 20e9  # Bandwidth in Hz (assuming 20 GHz)

# Generate random angles for clusters and paths

phi_r = np.random.uniform(0, 2 * np.pi, (N_C, N_P))

phi_t = np.random.uniform(0, 2 * np.pi, (N_C, N_P))

# Generate complex Gaussian channel gains

channel_gains = (np.random.randn(N_C, N_P) + 1j * np.random.randn(N_C, N_P)) / np.sqrt(2)

# Define antenna array response vectors

def array_response(angle, N):

    return (1 / np.sqrt(N)) * np.exp(1j * np.pi * np.arange(N) * np.sin(angle))

# RNN based hybrid Precoder

# Calculate the channel matrix H_k for each user

H_k = []

for k in range(K):

    H_k_user = np.zeros((N_R, N_T), dtype=complex)

    for i in range(N_C):

        for j in range(N_P):

            ar = array_response(phi_r[i, j], N_R)

            at = array_response(phi_t[i, j], N_T)

            H_k_user += channel_gains[i, j] * np.outer(ar, at)

    H_k_user *= np.sqrt((N_T * N_R) / (N_C * N_P))

    H_k.append(H_k_user)

# Use only the necessary elements from the predicted arrays

W_R = (w_R_real[:N_R * N_C] + 1j * w_R_imag[:N_R * N_C]).reshape(N_C, N_R).T

V_R = (V_R_real[:N_T * N_C] + 1j * V_R_imag[:N_T * N_C]).reshape(N_C, N_T).T

# Calculate the effective channel h_(l_k)

h_lk = []

for k in range(K):

    H_k_user = H_k[k]

    h_lk_user = np.conjugate(W_R).T @ H_k_user @ V_R

    h_lk.append(h_lk_user)

# Compute SINR for each user terminal for the original method

SINR = []

for k in range(K):

    numerator = (P / K) * np.abs(np.dot(h_lk[k], V_B_real[k] + 1j * V_B_imag[k]))**2

    denominator = np.sum([(P / K) * np.abs(np.dot(h_lk[j], V_B_real[j] + 1j * V_B_imag[j]))**2 for j in range(K) if j != k]) + sigma2

    SINR_val = numerator / denominator

    SINR.append(SINR_val)

# Convert SINR to a numpy array

SINR = np.array(SINR)

# Convert SINR from linear scale to dB scale

SINR_dB = 10 * np.log10(SINR)

# Calculate sumrate

sumrate = np.log2(1 + SINR)

# Calculate spectral efficiency by dividing sumrate by bandwidth

spectral_efficiency = sumrate / bandwidth

# MMSE PRECODER

# Initialize H_k matrix for MMSE precoder

H_k = np.zeros((K, N_R, N_T), dtype=complex)

# Compute H_k

for k in range(K):

    for i in range(N_C):

        for l in range(N_P):

            a_r = array_response_vector(phi_r[i, l], N_R)

            a_t = array_response_vector(phi_t[i, l], N_T)

            H_k[k] += np.sqrt((N_T * N_R) / (N_C * N_P)) * a_ik_il[i, l] * np.outer(a_r, a_t)

# Compute A_k, analog precoder v_R_k, B_K, G_k, and analog combiner w_R_k

A_k = np.zeros((K, N_T, N_R), dtype=complex)

v_R_k = np.zeros((K, N_T), dtype=complex)

B_K = np.zeros((K, N_R), dtype=complex)

G_k = np.zeros((K, N_R, N_R), dtype=complex)

w_R_k = np.zeros((K, N_R), dtype=complex)

for k in range(K):

    A_k[k] = np.linalg.inv(H_k[k].conj().T @ H_k[k] + (K * sigma2 / P) * np.eye(N_T)) @ H_k[k].conj().T

    A_k_phase_angles = np.angle(A_k[k]).flatten()

    v_R_k[k] = 1 / np.sqrt(N_T) * np.exp(1j * A_k_phase_angles[:N_T])

    B_K[k] = (H_k[k] @ v_R_k[k][:, np.newaxis]).flatten()

    G_k[k] = np.linalg.inv(B_K[k].conj().T @ B_K[k] + (K * sigma2 / P) * np.eye(N_R)) @ B_K[k].conj().T @ B_K[k]

    G_k_phase_angles = np.angle(G_k[k]).flatten()

    w_R_k[k] = 1 / np.sqrt(N_R) * np.exp(1j * G_k_phase_angles[:N_R])

# Compute the effective channel h_e_k

h_e_k = np.zeros((K, N_R), dtype=complex)

for k in range(K):

    h_e_k[k] = w_R_k[k].conj().T @ H_k[k] @ v_R_k[k]

# Compute the digital precoder V_B

H_e = np.array(h_e_k)

V_B = np.linalg.inv(H_e.conj().T @ H_e + (K * sigma2 / P) * np.eye(K)) @ H_e.conj().T

# Compute SINR in dB for each user terminal

SINR_dB = []

for k in range(K):

    numerator = (P / K) * np.abs(h_e_k[k] @ V_B[:, k])**2

    denominator = np.sum([(P / K) * np.abs(h_e_k[k] @ V_B[:, j])**2 for j in range(K) if j != k]) + sigma2

    SINR_k = numerator / denominator

    SINR_dB.append(10 * np.log10(SINR_k))

# Compute sum-rate for each user terminal

sum_rate = sum([np.log2(1 + 10**(SINR / 10)) for SINR in SINR_dB])

# Compute spectral efficiency per Hertz

spectral_efficiency_per_Hz = sum_rate / B

#zeroforcing

# Initialize H_k matrix for zero forcing

H_k = np.zeros((N_C, N_P), dtype=complex)

# Compute H_k

for i in range(N_C):

    for j in range(N_P):

        cos_term = np.cos(phi_r[i] - phi_t[j])

        H_k[i, j] = np.sqrt((N_T * N_R) / (N_C * N_P)) * channel_gains[i, j] * cos_term

# Zero Forcing Precoder

V_ZF = np.linalg.pinv(H_k)

# Normalize precoder

V_ZF = V_ZF / np.linalg.norm(V_ZF, axis=0)

# Effective channel

H_e = H_k @ V_ZF

# Compute SINR for each user terminal

SINR = []

for i in range(N_C):

    h_l_k = H_e[i, :]

    numerator = (p / sigma2) * np.abs(h_l_k)**2

    interference = np.sum([(p / sigma2) * np.abs(H_e[j, :])**2 for j in range(N_C) if j != i])

    denominator = interference + sigma2

    SINR_val = numerator / denominator

    SINR.append(SINR_val)

SINR = np.array(SINR)

# Compute achievable data rate (r_k) for each user terminal

r_k = np.log2(1 + SINR)

# Compute spectral efficiency (SE_k) for each user terminal

SE_k = r_k / B

# Compute sum-rate

sum_rate = np.sum(r_k)

# MRT Precoder

# Compute H_k

for i in range(N_C):

    for j in range(N_P):

        cos_term = np.cos(phi_r[i] - phi_t[j])

        H_k[i, j] = np.sqrt((N_T * N_R) / (N_C * N_P)) * channel_gains[i, j] * cos_term

# Maximum Ratio Transmission Precoder

V_MRT = H_k.conj().T

# Normalize precoder

V_MRT = V_MRT / np.linalg.norm(V_MRT, axis=0)

# Effective channel

H_e = H_k @ V_MRT

# Compute SINR for each user terminal

SINR = []

for i in range(N_C):

    h_l_k = H_e[i, :]

    numerator = (p / sigma2) * np.abs(h_l_k)**2

    interference = np.sum([(p / sigma2) * np.abs(H_e[j, :])**2 for j in range(N_C) if j != i])

    denominator = interference + sigma2

    SINR_val = numerator / denominator

    SINR.append(SINR_val)

SINR = np.array(SINR)

# Compute achievable data rate (r_k) for each user terminal

r_k = np.log2(1 + SINR)

# Compute spectral efficiency (SE_k) for each user terminal

SE_k = r_k / B

# Compute sum-rate

sum_rate = np.sum(r_k)

# Alamouti

# Initialize H_k matrix

H_k = np.zeros((N_C, N_P, N_T), dtype=complex)

# Compute H_k

for i in range(N_C):

    for j in range(N_P):

        cos_term = np.cos(phi_r[i] - phi_t[j])

        for k in range(N_T):

            H_k[i, j, k] = np.sqrt((N_T * N_R) / (N_C * N_P)) * channel_gains[i, j] * cos_term

# Alamouti precoding

V_Alamouti = np.zeros((N_T, 2), dtype=complex)

V_Alamouti[0, 0] = 1

V_Alamouti[1, 1] = 1

V_Alamouti[0, 1] = -1

V_Alamouti[1, 0] = 1

# Effective channel for Alamouti

H_e = np.zeros((N_C, 2), dtype=complex)

for i in range(N_C):

    H_e[i, :] = np.dot(H_k[i, :, :], V_Alamouti[:, 0]) + np.dot(H_k[i, :, :], V_Alamouti[:, 1])

# Compute SINR for each user terminal

SINR = []

for i in range(N_C):

    h_l_k = H_e[i, :]

    numerator = (p / sigma2) * np.abs(h_l_k)**2

    interference = np.sum([(p / sigma2) * np.abs(H_e[j, :])**2 for j in range(N_C) if j != i])

    denominator = interference + sigma2

    SINR_val = numerator / denominator

    SINR.append(SINR_val)

SINR = np.array(SINR)

# Compute achievable data rate (r_k) for each user terminal

r_k = np.log2(1 + SINR)

# Compute spectral efficiency (SE_k) for each user terminal

SE_k = r_k / B

# Compute sum-rate

sum_rate = np.sum(r_k)

# Plot Spectral Efficiency vs Number of RF Chains

plt.figure(figsize=(10, 6))

plt.plot(num_rf_chains, se_mmse_vals, marker='o', label='MMSE', markevery=0.25)

plt.plot(num_rf_chains, se_zf_vals, marker='s', label='ZF', markevery=0.25)  # Square marker

plt.plot(num_rf_chains, se_mrt_vals, marker='^', label='MRT', markevery=1)  # Triangle marker

plt.plot(num_rf_chains, se_alamouti_vals, marker='x', label='Alamouti', markevery=1)  # Diamond marker

plt.plot(num_rf_chains, se_ml_vals, marker='d', label='RNN-based', markevery=1)  # X marker

plt.xlabel('Number of RF Chains')

plt.ylabel('Spectral Efficiency (bps/Hz)')

plt.title('Spectral Efficiency vs Number of RF Chains')

plt.legend()

plt.grid(True, linestyle='--', linewidth=1)

plt.xticks(num_rf_chains)  # Set x-ticks to be whole numbers (4, 5, 6, 7, 8)

plt.show()

Spectral Efficiency vs Number of Transmitter Antennas

import pandas as pd

import numpy as np

import matplotlib.pyplot as plt

# Load the predicted results from the machine learning modelthat is saved in excel form

predicted_df = pd.read_excel('/content/drive/MyDrive/predictions_with_names.xlsx')

# Extract the predicted results (digital precoder Vb, analog precoder Vr and analog combiner Wr)

V_B_real = predicted_df['V_B_real'].values

V_B_imag = predicted_df['V_B_imag'].values

V_R_real = predicted_df['V_R_real'].values

V_R_imag = predicted_df['V_R_imag'].values

w_R_real = predicted_df['w_R_real'].values

w_R_imag = predicted_df['w_R_imag'].values

# Given parameters

N_R = 64

N_P = 5

N_C = 10

frequency = 1e12  # 1 THz in Hz

sigma2 = 1  # Noise variance

P = 10**(40 / 10)  # Transmit power in linear scale

B = 20e9  # Bandwidth in Hz (20 GHz)

# Generate random angles for clusters and paths

phi_r = np.random.uniform(0, 2 * np.pi, (N_C, N_P))

phi_t = np.random.uniform(0, 2 * np.pi, (N_C, N_P))

# Generate complex Gaussian channel gains

channel_gains = (np.random.randn(N_C, N_P) + 1j * np.random.randn(N_C, N_P)) / np.sqrt(2)

# Define antenna array response vectors

def array_response(angle, N):

    return (1 / np.sqrt(N)) * np.exp(1j * np.pi * np.arange(N) * np.sin(angle))

# Initialize lists to store results

se_mmse_vals = []

se_zf_vals = []

se_mrt_vals = []

se_alamouti_vals = []

se_ml_vals = []

# Loop over different numbers of transmitter antennas

num_tx_antennas = np.arange(1, 257)

for N_T in num_tx_antennas:

    # Calculate the channel matrix H_k for each user

    H_k = []

    for k in range(N_C):

        H_k_user = np.zeros((N_R, N_T), dtype=complex)

        for i in range(N_C):

            for j in range(N_P):

                ar = array_response(phi_r[i, j], N_R)

                at = array_response(phi_t[i, j], N_T)

                H_k_user += channel_gains[i, j] * np.outer(ar, at)

        H_k_user *= np.sqrt((N_T * N_R) / (N_C * N_P))

        H_k.append(H_k_user)

    # Use only the necessary elements from the predicted arrays

    W_R = (w_R_real[:N_R * N_C] + 1j * w_R_imag[:N_R * N_C]).reshape(N_C, N_R).T

    V_R = (V_R_real[:N_T * N_C] + 1j * V_R_imag[:N_T * N_C]).reshape(N_C, N_T).T

    # Calculate the effective channel h_(l_k)

    h_lk = []

    for k in range(N_C):

        H_k_user = H_k[k]

        h_lk_user = np.conjugate(W_R).T @ H_k_user @ V_R

        h_lk.append(h_lk_user)

    # Compute SINR for each user terminal for the original method

    SINR = []

    for k in range(N_C):

        numerator = (P / N_C) * np.abs(np.dot(h_lk[k], V_B_real[k] + 1j * V_B_imag[k]))**2

        denominator = np.sum([(P / N_C) * np.abs(np.dot(h_lk[j], V_B_real[j] + 1j * V_B_imag[j]))**2 for j in range(N_C) if j != k]) + sigma2

        SINR_val = numerator / denominator

        SINR.append(SINR_val)

    # Convert SINR to a numpy array

    SINR = np.array(SINR)

    # Convert SINR from linear scale to dB scale

    SINR_dB = 10 * np.log10(SINR)

    # Calculate sumrate

    sumrate = np.sum(np.log2(1 + SINR))

    # Calculate spectral efficiency by dividing sumrate by bandwidth

    se_ml = sumrate / B

    se_ml_vals.append(se_ml)

    # MMSE PRECODER

    # Initialize H_k matrix for MMSE precoder

    H_k_mmse = np.zeros((N_C, N_R, N_T), dtype=complex)

    # Compute H_k

    for k in range(N_C):

        for i in range(N_C):

            for l in range(N_P):

                ar = array_response(phi_r[i, l], N_R)

                at = array_response(phi_t[i, l], N_T)

                H_k_mmse[k] += np.sqrt((N_T * N_R) / (N_C * N_P)) * channel_gains[i, l] * np.outer(ar, at)

    # Compute A_k, analog precoder v_R_k, B_K, G_k, and analog combiner w_R_k

    A_k = np.zeros((N_C, N_T, N_R), dtype=complex)

    v_R_k = np.zeros((N_C, N_T), dtype=complex)

    B_K = np.zeros((N_C, N_R), dtype=complex)

    G_k = np.zeros((N_C, N_R, N_R), dtype=complex)

    w_R_k = np.zeros((N_C, N_R), dtype=complex)

    for k in range(N_C):

        A_k[k] = np.linalg.inv(H_k_mmse[k].conj().T @ H_k_mmse[k] + (N_C * sigma2 / P) * np.eye(N_T)) @ H_k_mmse[k].conj().T

        A_k_phase_angles = np.angle(A_k[k]).flatten()

        v_R_k[k] = 1 / np.sqrt(N_T) * np.exp(1j * A_k_phase_angles[:N_T])

        B_K[k] = (H_k_mmse[k] @ v_R_k[k][:, np.newaxis]).flatten()

        G_k[k] = np.linalg.inv(B_K[k].conj().T @ B_K[k] + (N_C * sigma2 / P) * np.eye(N_R)) @ B_K[k].conj().T @ B_K[k]

        G_k_phase_angles = np.angle(G_k[k]).flatten()

        w_R_k[k] = 1 / np.sqrt(N_R) * np.exp(1j * G_k_phase_angles[:N_R])

    # Compute the effective channel h_e_k

    h_e_k = np.zeros((N_C, N_R), dtype=complex)

    for k in range(N_C):

        h_e_k[k] = w_R_k[k].conj().T @ H_k_mmse[k] @ v_R_k[k]

    # Compute the digital precoder V_B

    H_e = np.array(h_e_k)

    V_B_mmse = np.linalg.inv(H_e.conj().T @ H_e + (N_C * sigma2 / P) * np.eye(N_C)) @ H_e.conj().T

    # Compute SINR in dB for each user terminal

    SINR_dB_mmse = []

    for k in range(N_C):

        numerator = (P / N_C) * np.abs(h_e_k[k] @ V_B_mmse[:, k])**2

        denominator = np.sum([(P / N_C) * np.abs(h_e_k[k] @ V_B_mmse[:, j])**2 for j in range(N_C) if j != k]) + sigma2

        SINR_k = numerator / denominator

        SINR_dB_mmse.append(10 * np.log10(SINR_k))

    # Compute sum-rate for each user terminal

    sum_rate_mmse = np.sum([np.log2(1 + 10**(SINR / 10)) for SINR in SINR_dB_mmse])

    # Compute spectral efficiency per Hertz

    spectral_efficiency_mmse = sum_rate_mmse / B

    se_mmse_vals.append(spectral_efficiency_mmse)

    # Zero Forcing Precoder

    V_ZF = np.linalg.pinv(H_e)

    # Normalize precoder

    V_ZF = V_ZF / np.linalg.norm(V_ZF, axis=0)

    # Effective channel

    H_e_zf = H_e @ V_ZF

    # Compute SINR for each user terminal

    SINR_zf = []

    for k in range(N_C):

        h_l_k = H_e_zf[k, :]

        numerator = (P / N_C) * np.abs(h_l_k)**2

        interference = np.sum([(P / N_C) * np.abs(H_e_zf[j, :])**2 for j in range(N_C) if j != k])

        denominator = interference + sigma2

        SINR_val = numerator / denominator

        SINR_zf.append(SINR_val)

    SINR_zf = np.array(SINR_zf)

    # Compute achievable data rate (r_k) for each user terminal

    r_k_zf = np.log2(1 + SINR_zf)

    # Compute spectral efficiency (SE_k) for each user terminal

    SE_k_zf = r_k_zf / B

    # Compute sum-rate for ZF

    sum_rate_zf = np.sum(r_k_zf)

    se_zf_vals.append(sum_rate_zf)

    # MRT Precoder

    V_MRT = H_e.conj().T

    # Normalize precoder

    V_MRT = V_MRT / np.linalg.norm(V_MRT, axis=0)

    # Effective channel

    H_e_mrt = H_e @ V_MRT

    # Compute SINR for each user terminal

    SINR_mrt = []

    for k in range(N_C):

        h_l_k = H_e_mrt[k, :]

        numerator = (P / N_C) * np.abs(h_l_k)**2

        interference = np.sum([(P / N_C) * np.abs(H_e_mrt[j, :])**2 for j in range(N_C) if j != k])

        denominator = interference + sigma2

        SINR_val = numerator / denominator

        SINR_mrt.append(SINR_val)

    SINR_mrt = np.array(SINR_mrt)

    # Compute achievable data rate (r_k) for each user terminal

    r_k_mrt = np.log2(1 + SINR_mrt)

    # Compute spectral efficiency (SE_k) for each user terminal

    SE_k_mrt = r_k_mrt / B

    # Compute sum-rate for MRT

    sum_rate_mrt = np.sum(r_k_mrt)

    se_mrt_vals.append(sum_rate_mrt)

    # Alamouti Precoding

    if N_T < 2:

        se_alamouti_vals.append(0)

        continue

    # Initialize H_k matrix

    H_k_alamouti = np.zeros((N_C, N_R, N_T), dtype=complex)

    # Compute H_k

    for k in range(N_C):

        for i in range(N_C):

            for j in range(N_P):

                ar = array_response(phi_r[i, j], N_R)

                at = array_response(phi_t[i, j], N_T)

                H_k_alamouti[k] += channel_gains[i, j] * np.outer(ar, at)

        H_k_alamouti[k] *= np.sqrt((N_T * N_R) / (N_C * N_P))

    # Alamouti precoding

    V_Alamouti = np.zeros((N_T, 2), dtype=complex)

    V_Alamouti[0, 0] = 1

    V_Alamouti[1, 1] = 1

    V_Alamouti[0, 1] = -1

    V_Alamouti[1, 0] = 1

    # Effective channel for Alamouti

    H_e_alamouti = np.zeros((N_C, 2), dtype=complex)

    for k in range(N_C):

        H_e_alamouti[k, :] = np.dot(H_k_alamouti[k, :, :], V_Alamouti[:, 0]) + np.dot(H_k_alamouti[k, :, :], V_Alamouti[:, 1])

    # Compute SINR for each user terminal

    SINR_alamouti = []

    for k in range(N_C):

        h_l_k = H_e_alamouti[k, :]

        numerator = (P / N_C) * np.abs(h_l_k)**2

        interference = np.sum([(P / N_C) * np.abs(H_e_alamouti[j, :])**2 for j in range(N_C) if j != k])

        denominator = interference + sigma2

        SINR_val = numerator / denominator

        SINR_alamouti.append(SINR_val)

    SINR_alamouti = np.array(SINR_alamouti)

    # Compute achievable data rate (r_k) for each user terminal

    r_k_alamouti = np.log2(1 + SINR_alamouti)

    # Compute spectral efficiency (SE_k) for each user terminal

    SE_k_alamouti = r_k_alamouti / B

    # Compute sum-rate for Alamouti

    sum_rate_alamouti = np.sum(r_k_alamouti)

    se_alamouti_vals.append(sum_rate_alamouti)

# Plot Spectral Efficiency vs Number of Transmitter Antennas

plt.figure(figsize=(10, 6))

plt.plot(num_tx_antennas, se_mmse_vals, label='MMSE', color='blue', marker='o', markevery=25)

plt.plot(num_tx_antennas, se_zf_vals, label='ZF', color='orange', marker='s', markevery=25)

plt.plot(num_tx_antennas, se_mrt_vals, label='MRT', color='green', marker='^', markevery=25)

plt.plot(num_tx_antennas, se_alamouti_vals, label='Alamouti', color='red', marker='d', markevery=25)

plt.plot(num_tx_antennas, se_ml_vals, label='RNN-based', color='purple', marker='x', markevery=25)

plt.xlabel('Number of Transmitter Antennas')

plt.ylabel('Spectral Efficiency (bps/Hz)')

plt.title('Spectral Efficiency vs Number of Transmitter Antennas')

plt.legend()

plt.grid(True, linestyle='--', linewidth=0.5)

plt.xlim(0, 256)

plt.show()

BER vs SNR for 16-QAM for Nt=256

import pandas as pd

import numpy as np

import matplotlib.pyplot as plt

# Load the predicted results from the machine learning modelthat is saved in excel form

predicted_df = pd.read_excel('/content/drive/MyDrive/predictions_with_names.xlsx')

#  the predicted results contain columns 'V_B_real', 'V_B_imag', 'V_R_real', 'V_R_imag', 'w_R_real', 'w_R_imag' (digital precoder Vb, analog precoder Vr and analog combiner Wr)

V_B_real = predicted_df['V_B_real'].values

V_B_imag = predicted_df['V_B_imag'].values

V_R_real = predicted_df['V_R_real'].values

V_R_imag = predicted_df['V_R_imag'].values

w_R_real = predicted_df['w_R_real'].values

w_R_imag = predicted_df['w_R_imag'].values

# Given parameters

N_T = 64

N_R = 64

N_P = 5

N_C = 10

frequency = 1e12  # 1 THz in Hz

sigma2 = 10**(-10)  # Noise variance

K = 5

P = 10**(40 / 10)  # Transmit power in linear scale

sigma2 = 1

p = 10

B = 20e9  # Bandwidth in GHz (assuming 20 GHz)

# Generate random angles for clusters and paths

phi_r = np.random.uniform(0, 2 * np.pi, (N_C, N_P))

phi_t = np.random.uniform(0, 2 * np.pi, (N_C, N_P))

# Generate complex Gaussian channel gains

channel_gains = (np.random.randn(N_C, N_P) + 1j * np.random.randn(N_C, N_P)) / np.sqrt(2)

# Define antenna array response vectors

def array_response(angle, N):

    return (1 / np.sqrt(N)) * np.exp(1j * np.pi * np.arange(N) * np.sin(angle))

# RNN based hybrid Precoder

# Calculate the channel matrix H_k for each user

H_k = []

for k in range(K):

    H_k_user = np.zeros((N_R, N_T), dtype=complex)

    for i in range(N_C):

        for j in range(N_P):

            ar = array_response(phi_r[i, j], N_R)

            at = array_response(phi_t[i, j], N_T)

            H_k_user += channel_gains[i, j] * np.outer(ar, at)

    H_k_user *= np.sqrt((N_T * N_R) / (N_C * N_P))

    H_k.append(H_k_user)

# Use only the necessary elements from the predicted arrays

W_R = (w_R_real[:N_R * N_C] + 1j * w_R_imag[:N_R * N_C]).reshape(N_C, N_R).T

V_R = (V_R_real[:N_T * N_C] + 1j * V_R_imag[:N_T * N_C]).reshape(N_C, N_T).T

# Calculate the effective channel h_(l_k)

h_lk = []

for k in range(K):

    H_k_user = H_k[k]

    h_lk_user = np.conjugate(W_R).T @ H_k_user @ V_R

    h_lk.append(h_lk_user)

# Compute SINR for each user terminal for the original method

SINR = []

for k in range(K):

    numerator = (P / K) * np.abs(np.dot(h_lk[k], V_B_real[k] + 1j * V_B_imag[k]))**2

    denominator = np.sum([(P / K) * np.abs(np.dot(h_lk[j], V_B_real[j] + 1j * V_B_imag[j]))**2 for j in range(K) if j != k]) + sigma2

    SINR_val = numerator / denominator

    SINR.append(SINR_val)

# Convert SINR to a numpy array

SINR = np.array(SINR)

# Convert SINR from linear scale to dB scale

SINR_dB = 10 * np.log10(SINR)

# MMSE PRECODER

# Initialize H_k matrix for MMSE precoder

H_k = np.zeros((K, N_R, N_T), dtype=complex)

# Compute H_k

for k in range(K):

    for i in range(N_C):

        for l in range(N_P):

            a_r = array_response_vector(phi_r[i, l], N_R)

            a_t = array_response_vector(phi_t[i, l], N_T)

            H_k[k] += np.sqrt((N_T * N_R) / (N_C * N_P)) * a_ik_il[i, l] * np.outer(a_r, a_t)

# Compute A_k, analog precoder v_R_k, B_K, G_k, and analog combiner w_R_k

A_k = np.zeros((K, N_T, N_R), dtype=complex)

v_R_k = np.zeros((K, N_T), dtype=complex)

B_K = np.zeros((K, N_R), dtype=complex)

G_k = np.zeros((K, N_R, N_R), dtype=complex)

w_R_k = np.zeros((K, N_R), dtype=complex)

for k in range(K):

    A_k[k] = np.linalg.inv(H_k[k].conj().T @ H_k[k] + (K * sigma2 / P) * np.eye(N_T)) @ H_k[k].conj().T

    A_k_phase_angles = np.angle(A_k[k]).flatten()

    v_R_k[k] = 1 / np.sqrt(N_T) * np.exp(1j * A_k_phase_angles[:N_T])

    B_K[k] = (H_k[k] @ v_R_k[k][:, np.newaxis]).flatten()

    G_k[k] = np.linalg.inv(B_K[k].conj().T @ B_K[k] + (K * sigma2 / P) * np.eye(N_R)) @ B_K[k].conj().T @ B_K[k]

    G_k_phase_angles = np.angle(G_k[k]).flatten()

    w_R_k[k] = 1 / np.sqrt(N_R) * np.exp(1j * G_k_phase_angles[:N_R])

# Compute the effective channel h_e_k

h_e_k = np.zeros((K, N_R), dtype=complex)

for k in range(K):

    h_e_k[k] = w_R_k[k].conj().T @ H_k[k] @ v_R_k[k]

# Compute the digital precoder V_B

H_e = np.array(h_e_k)

V_B = np.linalg.inv(H_e.conj().T @ H_e + (K * sigma2 / P) * np.eye(K)) @ H_e.conj().T

# Compute SINR in dB for each user terminal

SINR_dB = []

for k in range(K):

    numerator = (P / K) * np.abs(h_e_k[k] @ V_B[:, k])**2

    denominator = np.sum([(P / K) * np.abs(h_e_k[k] @ V_B[:, j])**2 for j in range(K) if j != k]) + sigma2

    SINR_k = numerator / denominator

    SINR_dB.append(10 * np.log10(SINR_k))

#zeroforcing

# Initialize H_k matrix for zero forcing

H_k = np.zeros((N_C, N_P), dtype=complex)

# Compute H_k

for i in range(N_C):

    for j in range(N_P):

        cos_term = np.cos(phi_r[i] - phi_t[j])

        H_k[i, j] = np.sqrt((N_T * N_R) / (N_C * N_P)) * channel_gains[i, j] * cos_term

# Zero Forcing Precoder

V_ZF = np.linalg.pinv(H_k)

# Normalize precoder

V_ZF = V_ZF / np.linalg.norm(V_ZF, axis=0)

# Effective channel

H_e = H_k @ V_ZF

# Compute SINR for each user terminal

SINR = []

for i in range(N_C):

    h_l_k = H_e[i, :]

    numerator = (p / sigma2) * np.abs(h_l_k)**2

    interference = np.sum([(p / sigma2) * np.abs(H_e[j, :])**2 for j in range(N_C) if j != i])

    denominator = interference + sigma2

    SINR_val = numerator / denominator

    SINR.append(SINR_val)

SINR = np.array(SINR)

# MRT Precoder

# Compute H_k

for i in range(N_C):

    for j in range(N_P):

        cos_term = np.cos(phi_r[i] - phi_t[j])

        H_k[i, j] = np.sqrt((N_T * N_R) / (N_C * N_P)) * channel_gains[i, j] * cos_term

# Maximum Ratio Transmission Precoder

V_MRT = H_k.conj().T

# Normalize precoder

V_MRT = V_MRT / np.linalg.norm(V_MRT, axis=0)

# Effective channel

H_e = H_k @ V_MRT

# Compute SINR for each user terminal

SINR = []

for i in range(N_C):

    h_l_k = H_e[i, :]

    numerator = (p / sigma2) * np.abs(h_l_k)**2

    interference = np.sum([(p / sigma2) * np.abs(H_e[j, :])**2 for j in range(N_C) if j != i])

    denominator = interference + sigma2

    SINR_val = numerator / denominator

    SINR.append(SINR_val)

SINR = np.array(SINR)

# Alamouti

# Initialize H_k matrix

H_k = np.zeros((N_C, N_P, N_T), dtype=complex)

# Compute H_k

for i in range(N_C):

    for j in range(N_P):

        cos_term = np.cos(phi_r[i] - phi_t[j])

        for k in range(N_T):

            H_k[i, j, k] = np.sqrt((N_T * N_R) / (N_C * N_P)) * channel_gains[i, j] * cos_term

# Alamouti precoding

V_Alamouti = np.zeros((N_T, 2), dtype=complex)

V_Alamouti[0, 0] = 1

V_Alamouti[1, 1] = 1

V_Alamouti[0, 1] = -1

V_Alamouti[1, 0] = 1

# Effective channel for Alamouti

H_e = np.zeros((N_C, 2), dtype=complex)

for i in range(N_C):

    H_e[i, :] = np.dot(H_k[i, :, :], V_Alamouti[:, 0]) + np.dot(H_k[i, :, :], V_Alamouti[:, 1])

# Compute SINR for each user terminal

SINR = []

for i in range(N_C):

    h_l_k = H_e[i, :]

    numerator = (p / sigma2) * np.abs(h_l_k)**2

    interference = np.sum([(p / sigma2) * np.abs(H_e[j, :])**2 for j in range(N_C) if j != i])

    denominator = interference + sigma2

    SINR_val = numerator / denominator

    SINR.append(SINR_val)

SINR = np.array(SINR)

def calculate_ber(sinr):

    ber = (3/8) * erfc(math.sqrt((3/2) * (sinr / (1 + sinr))))

    return ber

# Plot BER vs SNR

plt.figure(figsize=(10, 6))

plt.semilogy(snr_db, ber_mmse_vals, label='MMSE', marker='o')

plt.semilogy(snr_db, ber_zf_vals, label='ZF', marker='x')

plt.semilogy(snr_db, ber_mrt_vals, label='MRT', marker='s')

plt.semilogy(snr_db, ber_alamouti_vals, label='Alamouti', marker='d')

plt.semilogy(snr_db, ber_ml_vals, label='RNN-based', marker='^')

plt.xlabel('SNR (dB)')

plt.ylabel('BER')

plt.title('BER vs SNR for 16-QAM for Nt=256')

plt.legend()

plt.grid(True, which='both', linestyle='--', linewidth=0.5)

plt.show()

BER vs SNR for 64-QAM for Nt=256

import pandas as pd

import numpy as np

import matplotlib.pyplot as plt

# Load the predicted results from the machine learning modelthat is saved in excel form

predicted_df = pd.read_excel('/content/drive/MyDrive/predictions_with_names.xlsx')

#  the predicted results contain columns 'V_B_real', 'V_B_imag', 'V_R_real', 'V_R_imag', 'w_R_real', 'w_R_imag'(digital precoder Vb, analog precoder Vr and analog combiner Wr)

V_B_real = predicted_df['V_B_real'].values

V_B_imag = predicted_df['V_B_imag'].values

V_R_real = predicted_df['V_R_real'].values

V_R_imag = predicted_df['V_R_imag'].values

w_R_real = predicted_df['w_R_real'].values

w_R_imag = predicted_df['w_R_imag'].values

# Given parameters

N_T = 256

N_R = 64

N_P = 5

N_C = 10

frequency = 1e12  # 1 THz in Hz

sigma2 = 10**(-10)  # Noise variance

K = 5

P = 10**(40 / 10)  # Transmit power in linear scale

sigma2 = 1

p = 10

B = 20e9  # Bandwidth in GHz (assuming 20 GHz)

# Generate random angles for clusters and paths

phi_r = np.random.uniform(0, 2 * np.pi, (N_C, N_P))

phi_t = np.random.uniform(0, 2 * np.pi, (N_C, N_P))

# Generate complex Gaussian channel gains

channel_gains = (np.random.randn(N_C, N_P) + 1j * np.random.randn(N_C, N_P)) / np.sqrt(2)

# Define antenna array response vectors

def array_response(angle, N):

    return (1 / np.sqrt(N)) * np.exp(1j * np.pi * np.arange(N) * np.sin(angle))

# RNN based hybrid Precoder

# Calculate the channel matrix H_k for each user

H_k = []

for k in range(K):

    H_k_user = np.zeros((N_R, N_T), dtype=complex)

    for i in range(N_C):

        for j in range(N_P):

            ar = array_response(phi_r[i, j], N_R)

            at = array_response(phi_t[i, j], N_T)

            H_k_user += channel_gains[i, j] * np.outer(ar, at)

    H_k_user *= np.sqrt((N_T * N_R) / (N_C * N_P))

    H_k.append(H_k_user)

# Use only the necessary elements from the predicted arrays

W_R = (w_R_real[:N_R * N_C] + 1j * w_R_imag[:N_R * N_C]).reshape(N_C, N_R).T

V_R = (V_R_real[:N_T * N_C] + 1j * V_R_imag[:N_T * N_C]).reshape(N_C, N_T).T

# Calculate the effective channel h_(l_k)

h_lk = []

for k in range(K):

    H_k_user = H_k[k]

    h_lk_user = np.conjugate(W_R).T @ H_k_user @ V_R

    h_lk.append(h_lk_user)

# Compute SINR for each user terminal for the original method

SINR = []

for k in range(K):

    numerator = (P / K) * np.abs(np.dot(h_lk[k], V_B_real[k] + 1j * V_B_imag[k]))**2

    denominator = np.sum([(P / K) * np.abs(np.dot(h_lk[j], V_B_real[j] + 1j * V_B_imag[j]))**2 for j in range(K) if j != k]) + sigma2

    SINR_val = numerator / denominator

    SINR.append(SINR_val)

# Convert SINR to a numpy array

SINR = np.array(SINR)

# Convert SINR from linear scale to dB scale

SINR_dB = 10 * np.log10(SINR)

# MMSE PRECODER

# Initialize H_k matrix for MMSE precoder

H_k = np.zeros((K, N_R, N_T), dtype=complex)

# Compute H_k

for k in range(K):

    for i in range(N_C):

        for l in range(N_P):

            a_r = array_response_vector(phi_r[i, l], N_R)

            a_t = array_response_vector(phi_t[i, l], N_T)

            H_k[k] += np.sqrt((N_T * N_R) / (N_C * N_P)) * a_ik_il[i, l] * np.outer(a_r, a_t)

# Compute A_k, analog precoder v_R_k, B_K, G_k, and analog combiner w_R_k

A_k = np.zeros((K, N_T, N_R), dtype=complex)

v_R_k = np.zeros((K, N_T), dtype=complex)

B_K = np.zeros((K, N_R), dtype=complex)

G_k = np.zeros((K, N_R, N_R), dtype=complex)

w_R_k = np.zeros((K, N_R), dtype=complex)

for k in range(K):

    A_k[k] = np.linalg.inv(H_k[k].conj().T @ H_k[k] + (K * sigma2 / P) * np.eye(N_T)) @ H_k[k].conj().T

    A_k_phase_angles = np.angle(A_k[k]).flatten()

    v_R_k[k] = 1 / np.sqrt(N_T) * np.exp(1j * A_k_phase_angles[:N_T])

    B_K[k] = (H_k[k] @ v_R_k[k][:, np.newaxis]).flatten()

    G_k[k] = np.linalg.inv(B_K[k].conj().T @ B_K[k] + (K * sigma2 / P) * np.eye(N_R)) @ B_K[k].conj().T @ B_K[k]

    G_k_phase_angles = np.angle(G_k[k]).flatten()

    w_R_k[k] = 1 / np.sqrt(N_R) * np.exp(1j * G_k_phase_angles[:N_R])

# Compute the effective channel h_e_k

h_e_k = np.zeros((K, N_R), dtype=complex)

for k in range(K):

    h_e_k[k] = w_R_k[k].conj().T @ H_k[k] @ v_R_k[k]

# Compute the digital precoder V_B

H_e = np.array(h_e_k)

V_B = np.linalg.inv(H_e.conj().T @ H_e + (K * sigma2 / P) * np.eye(K)) @ H_e.conj().T

# Compute SINR in dB for each user terminal

SINR_dB = []

for k in range(K):

    numerator = (P / K) * np.abs(h_e_k[k] @ V_B[:, k])**2

    denominator = np.sum([(P / K) * np.abs(h_e_k[k] @ V_B[:, j])**2 for j in range(K) if j != k]) + sigma2

    SINR_k = numerator / denominator

    SINR_dB.append(10 * np.log10(SINR_k))

#zeroforcing

# Initialize H_k matrix for zero forcing

H_k = np.zeros((N_C, N_P), dtype=complex)

# Compute H_k

for i in range(N_C):

    for j in range(N_P):

        cos_term = np.cos(phi_r[i] - phi_t[j])

        H_k[i, j] = np.sqrt((N_T * N_R) / (N_C * N_P)) * channel_gains[i, j] * cos_term

# Zero Forcing Precoder

V_ZF = np.linalg.pinv(H_k)

# Normalize precoder

V_ZF = V_ZF / np.linalg.norm(V_ZF, axis=0)

# Effective channel

H_e = H_k @ V_ZF

# Compute SINR for each user terminal

SINR = []

for i in range(N_C):

    h_l_k = H_e[i, :]

    numerator = (p / sigma2) * np.abs(h_l_k)**2

    interference = np.sum([(p / sigma2) * np.abs(H_e[j, :])**2 for j in range(N_C) if j != i])

    denominator = interference + sigma2

    SINR_val = numerator / denominator

    SINR.append(SINR_val)

SINR = np.array(SINR)

# MRT Precoder

# Compute H_k

for i in range(N_C):

    for j in range(N_P):

        cos_term = np.cos(phi_r[i] - phi_t[j])

        H_k[i, j] = np.sqrt((N_T * N_R) / (N_C * N_P)) * channel_gains[i, j] * cos_term

# Maximum Ratio Transmission Precoder

V_MRT = H_k.conj().T

# Normalize precoder

V_MRT = V_MRT / np.linalg.norm(V_MRT, axis=0)

# Effective channel

H_e = H_k @ V_MRT

# Compute SINR for each user terminal

SINR = []

for i in range(N_C):

    h_l_k = H_e[i, :]

    numerator = (p / sigma2) * np.abs(h_l_k)**2

    interference = np.sum([(p / sigma2) * np.abs(H_e[j, :])**2 for j in range(N_C) if j != i])

    denominator = interference + sigma2

    SINR_val = numerator / denominator

    SINR.append(SINR_val)

SINR = np.array(SINR)

# Alamouti

# Initialize H_k matrix

H_k = np.zeros((N_C, N_P, N_T), dtype=complex)

# Compute H_k

for i in range(N_C):

    for j in range(N_P):

        cos_term = np.cos(phi_r[i] - phi_t[j])

        for k in range(N_T):

            H_k[i, j, k] = np.sqrt((N_T * N_R) / (N_C * N_P)) * channel_gains[i, j] * cos_term

# Alamouti precoding

V_Alamouti = np.zeros((N_T, 2), dtype=complex)

V_Alamouti[0, 0] = 1

V_Alamouti[1, 1] = 1

V_Alamouti[0, 1] = -1

V_Alamouti[1, 0] = 1

# Effective channel for Alamouti

H_e = np.zeros((N_C, 2), dtype=complex)

for i in range(N_C):

    H_e[i, :] = np.dot(H_k[i, :, :], V_Alamouti[:, 0]) + np.dot(H_k[i, :, :], V_Alamouti[:, 1])

# Compute SINR for each user terminal

SINR = []

for i in range(N_C):

    h_l_k = H_e[i, :]

    numerator = (p / sigma2) * np.abs(h_l_k)**2

    interference = np.sum([(p / sigma2) * np.abs(H_e[j, :])**2 for j in range(N_C) if j != i])

    denominator = interference + sigma2

    SINR_val = numerator / denominator

    SINR.append(SINR_val)

SINR = np.array(SINR)

def calculate_ber_64qam(sinr):

    M = 64

    ber = (7/6) * (1 - (1/math.sqrt(M))) * erfc(math.sqrt((3 * math.log2(M) * sinr) / ((M - 1) * 2)))

    return ber

    # Plot BER vs SNR

plt.figure(figsize=(10, 6))

plt.semilogy(snr_db, ber_mmse_vals, label='MMSE', marker='o')

plt.semilogy(snr_db, ber_zf_vals, label='ZF', marker='x')

plt.semilogy(snr_db, ber_mrt_vals, label='MRT', marker='s')

plt.semilogy(snr_db, ber_alamouti_vals, label='Alamouti', marker='d')

plt.semilogy(snr_db, ber_ml_vals, label='RNN-based', marker='^')

plt.xlabel('SNR (dB)')

plt.ylabel('BER')

plt.title('BER vs SNR for 64-QAM for Nt=256')

plt.legend()

plt.grid(True, which='both', linestyle='--', linewidth=0.5)

plt.show()

BER vs SNR for 64-QAM for Nt=64

import pandas as pd

import numpy as np

import matplotlib.pyplot as plt

# Load the predicted results from the machine learning modelthat is saved in excel form

predicted_df = pd.read_excel('/content/drive/MyDrive/predictions_with_names.xlsx')

#  the predicted results contain columns 'V_B_real', 'V_B_imag', 'V_R_real', 'V_R_imag', 'w_R_real', 'w_R_imag' (digital precoder Vb, analog precoder Vr and analog combiner Wr)

V_B_real = predicted_df['V_B_real'].values

V_B_imag = predicted_df['V_B_imag'].values

V_R_real = predicted_df['V_R_real'].values

V_R_imag = predicted_df['V_R_imag'].values

w_R_real = predicted_df['w_R_real'].values

w_R_imag = predicted_df['w_R_imag'].values

# Given parameters

N_T = 64

N_P = 5

N_C = 10

frequency = 1e12  # 1 THz in Hz

sigma2 = 10**(-10)  # Noise variance

K = 5

P = 10**(40 / 10)  # Transmit power in linear scale

sigma2 = 1

p = 10

B = 20e9  # Bandwidth in GHz (assuming 20 GHz)

# Generate random angles for clusters and paths

phi_r = np.random.uniform(0, 2 * np.pi, (N_C, N_P))

phi_t = np.random.uniform(0, 2 * np.pi, (N_C, N_P))

# Generate complex Gaussian channel gains

channel_gains = (np.random.randn(N_C, N_P) + 1j * np.random.randn(N_C, N_P)) / np.sqrt(2)

# Define antenna array response vectors

def array_response(angle, N):

    return (1 / np.sqrt(N)) * np.exp(1j * np.pi * np.arange(N) * np.sin(angle))

# RNN based hybrid Precoder

# Calculate the channel matrix H_k for each user

H_k = []

for k in range(K):

    H_k_user = np.zeros((N_R, N_T), dtype=complex)

    for i in range(N_C):

        for j in range(N_P):

            ar = array_response(phi_r[i, j], N_R)

            at = array_response(phi_t[i, j], N_T)

            H_k_user += channel_gains[i, j] * np.outer(ar, at)

    H_k_user *= np.sqrt((N_T * N_R) / (N_C * N_P))

    H_k.append(H_k_user)

# Use only the necessary elements from the predicted arrays

W_R = (w_R_real[:N_R * N_C] + 1j * w_R_imag[:N_R * N_C]).reshape(N_C, N_R).T

V_R = (V_R_real[:N_T * N_C] + 1j * V_R_imag[:N_T * N_C]).reshape(N_C, N_T).T

# Calculate the effective channel h_(l_k)

h_lk = []

for k in range(K):

    H_k_user = H_k[k]

    h_lk_user = np.conjugate(W_R).T @ H_k_user @ V_R

    h_lk.append(h_lk_user)

# Compute SINR for each user terminal for the original method

SINR = []

for k in range(K):

    numerator = (P / K) * np.abs(np.dot(h_lk[k], V_B_real[k] + 1j * V_B_imag[k]))**2

    denominator = np.sum([(P / K) * np.abs(np.dot(h_lk[j], V_B_real[j] + 1j * V_B_imag[j]))**2 for j in range(K) if j != k]) + sigma2

    SINR_val = numerator / denominator

    SINR.append(SINR_val)

# Convert SINR to a numpy array

SINR = np.array(SINR)

# Convert SINR from linear scale to dB scale

SINR_dB = 10 * np.log10(SINR)

# MMSE PRECODER

# Initialize H_k matrix for MMSE precoder

H_k = np.zeros((K, N_R, N_T), dtype=complex)

# Compute H_k

for k in range(K):

    for i in range(N_C):

        for l in range(N_P):

            a_r = array_response_vector(phi_r[i, l], N_R)

            a_t = array_response_vector(phi_t[i, l], N_T)

            H_k[k] += np.sqrt((N_T * N_R) / (N_C * N_P)) * a_ik_il[i, l] * np.outer(a_r, a_t)

# Compute A_k, analog precoder v_R_k, B_K, G_k, and analog combiner w_R_k

A_k = np.zeros((K, N_T, N_R), dtype=complex)

v_R_k = np.zeros((K, N_T), dtype=complex)

B_K = np.zeros((K, N_R), dtype=complex)

G_k = np.zeros((K, N_R, N_R), dtype=complex)

w_R_k = np.zeros((K, N_R), dtype=complex)

for k in range(K):

    A_k[k] = np.linalg.inv(H_k[k].conj().T @ H_k[k] + (K * sigma2 / P) * np.eye(N_T)) @ H_k[k].conj().T

    A_k_phase_angles = np.angle(A_k[k]).flatten()

    v_R_k[k] = 1 / np.sqrt(N_T) * np.exp(1j * A_k_phase_angles[:N_T])

    B_K[k] = (H_k[k] @ v_R_k[k][:, np.newaxis]).flatten()

    G_k[k] = np.linalg.inv(B_K[k].conj().T @ B_K[k] + (K * sigma2 / P) * np.eye(N_R)) @ B_K[k].conj().T @ B_K[k]

    G_k_phase_angles = np.angle(G_k[k]).flatten()

    w_R_k[k] = 1 / np.sqrt(N_R) * np.exp(1j * G_k_phase_angles[:N_R])

# Compute the effective channel h_e_k

h_e_k = np.zeros((K, N_R), dtype=complex)

for k in range(K):

    h_e_k[k] = w_R_k[k].conj().T @ H_k[k] @ v_R_k[k]

# Compute the digital precoder V_B

H_e = np.array(h_e_k)

V_B = np.linalg.inv(H_e.conj().T @ H_e + (K * sigma2 / P) * np.eye(K)) @ H_e.conj().T

# Compute SINR in dB for each user terminal

SINR_dB = []

for k in range(K):

    numerator = (P / K) * np.abs(h_e_k[k] @ V_B[:, k])**2

    denominator = np.sum([(P / K) * np.abs(h_e_k[k] @ V_B[:, j])**2 for j in range(K) if j != k]) + sigma2

    SINR_k = numerator / denominator

    SINR_dB.append(10 * np.log10(SINR_k))

#zeroforcing

# Initialize H_k matrix for zero forcing

H_k = np.zeros((N_C, N_P), dtype=complex)

# Compute H_k

for i in range(N_C):

    for j in range(N_P):

        cos_term = np.cos(phi_r[i] - phi_t[j])

        H_k[i, j] = np.sqrt((N_T * N_R) / (N_C * N_P)) * channel_gains[i, j] * cos_term

# Zero Forcing Precoder

V_ZF = np.linalg.pinv(H_k)

# Normalize precoder

V_ZF = V_ZF / np.linalg.norm(V_ZF, axis=0)

# Effective channel

H_e = H_k @ V_ZF

# Compute SINR for each user terminal

SINR = []

for i in range(N_C):

    h_l_k = H_e[i, :]

    numerator = (p / sigma2) * np.abs(h_l_k)**2

    interference = np.sum([(p / sigma2) * np.abs(H_e[j, :])**2 for j in range(N_C) if j != i])

    denominator = interference + sigma2

    SINR_val = numerator / denominator

    SINR.append(SINR_val)

SINR = np.array(SINR)

# MRT Precoder

# Compute H_k

for i in range(N_C):

    for j in range(N_P):

        cos_term = np.cos(phi_r[i] - phi_t[j])

        H_k[i, j] = np.sqrt((N_T * N_R) / (N_C * N_P)) * channel_gains[i, j] * cos_term

# Maximum Ratio Transmission Precoder

V_MRT = H_k.conj().T

# Normalize precoder

V_MRT = V_MRT / np.linalg.norm(V_MRT, axis=0)

# Effective channel

H_e = H_k @ V_MRT

# Compute SINR for each user terminal

SINR = []

for i in range(N_C):

    h_l_k = H_e[i, :]

    numerator = (p / sigma2) * np.abs(h_l_k)**2

    interference = np.sum([(p / sigma2) * np.abs(H_e[j, :])**2 for j in range(N_C) if j != i])

    denominator = interference + sigma2

    SINR_val = numerator / denominator

    SINR.append(SINR_val)

SINR = np.array(SINR)

# Alamouti

# Initialize H_k matrix

H_k = np.zeros((N_C, N_P, N_T), dtype=complex)

# Compute H_k

for i in range(N_C):

    for j in range(N_P):

        cos_term = np.cos(phi_r[i] - phi_t[j])

        for k in range(N_T):

            H_k[i, j, k] = np.sqrt((N_T * N_R) / (N_C * N_P)) * channel_gains[i, j] * cos_term

# Alamouti precoding

V_Alamouti = np.zeros((N_T, 2), dtype=complex)

V_Alamouti[0, 0] = 1

V_Alamouti[1, 1] = 1

V_Alamouti[0, 1] = -1

V_Alamouti[1, 0] = 1

# Effective channel for Alamouti

H_e = np.zeros((N_C, 2), dtype=complex)

for i in range(N_C):

    H_e[i, :] = np.dot(H_k[i, :, :], V_Alamouti[:, 0]) + np.dot(H_k[i, :, :], V_Alamouti[:, 1])

# Compute SINR for each user terminal

SINR = []

for i in range(N_C):

    h_l_k = H_e[i, :]

    numerator = (p / sigma2) * np.abs(h_l_k)**2

    interference = np.sum([(p / sigma2) * np.abs(H_e[j, :])**2 for j in range(N_C) if j != i])

    denominator = interference + sigma2

    SINR_val = numerator / denominator

    SINR.append(SINR_val)

SINR = np.array(SINR)

def calculate_ber_64qam(sinr):

    M = 64

    ber = (7/6) * (1 - (1/math.sqrt(M))) * erfc(math.sqrt((3 * math.log2(M) * sinr) / ((M - 1) * 2)))

    return ber

    # Plot BER vs SNR

plt.figure(figsize=(10, 6))

plt.semilogy(snr_db, ber_mmse_vals, label='MMSE', marker='o')

plt.semilogy(snr_db, ber_zf_vals, label='ZF', marker='x')

plt.semilogy(snr_db, ber_mrt_vals, label='MRT', marker='s')

plt.semilogy(snr_db, ber_alamouti_vals, label='Alamouti', marker='d')

plt.semilogy(snr_db, ber_ml_vals, label='RNN-based', marker='^')

plt.xlabel('SNR (dB)')

plt.ylabel('BER')

plt.title('BER vs SNR for 64-QAM for Nt=64')

plt.legend()

plt.grid(True, which='both', linestyle='--', linewidth=0.5)

plt.show()
