# Supplementary material for: Spectral efficiency and BER analysis of RNN based hybrid precoding for cell free massive MIMO under terahertz communication
Source: PLoS One. 2025 Aug 14;20(8):e0328499. doi: 10.1371/journal.pone.0328499 (PMC12352664; doi:10.1371/journal.pone.0328499)
Supplement: Attachment — Submitted filename: RNN-Reviewer-Response PLOS Dec 24.pdf [file pone.0328499.s004.pdf]

Academic Editor’s comment

Note that changes made are shown in red in the revised manuscript.

Thank you very much for providing us with a constructive comment. We have addressed the comment as follows:

Academic Editor’s comment 1: Notably, the Rayleigh channel model remains inadequate for the THz range scenarios discussed, with no substantial updates to the mathematical modeling. **Authors’ reply to comment 1:** We agree with the Academic Editor’s comment. In an effort to improve the overall flow, we tried to clear the comment. Kindly see sub-section 3.1, page 7. as referred by [Reference 28]

Academic Editor’s comment 2: The BER curves still deviate from established literature, exhibiting exponential rather than expected linear decay for detectors like ZF and MMSE.

Authors’ reply to comment 2: We appreciate the Academic Editor’s insightful comment. To enhance the overall flow and clarity of the manuscript, we have addressed the comment as suggested. Kindly refer to Section 7, starting from page 18-20, for the revised Figure and description of Fig 7,8 and 9.

Author action: We re-evaluated the system model and simulation parameters to ensure the accuracy of the BER calculations by refining the channel model and adjusting SNR range to align with those used in comparable literature. The implementation of ZF and MMSE algorithms was verified to ensure compliance with theoretical models. Additional simulations were conducted to observe BER behavior over an extended SNR range. The revised BER results were cross-validated against established references, confirming the correction of previously observed exponential behavior to the expected linear decay. These updates were incorporated into the manuscript by revising Figures 7, 8, and 9 to reflect the corrected BER curves, enhancing the discussion in Section 7 to detail the methodological adjustments and their alignment with the literature, and emphasizing the significance of achieving the expected decay behavior for the accuracy of the presented results.

Academic Editor’s comment 3: Key phenomena affecting THz communications are mentioned but not integrated into the model or numerical results.

Authors' reply to comment 3: We appreciate the Academic Editor's insightful comment. To enhance the overall flow and clarity of the manuscript, we have addressed the comment as suggested. Kindly refer to Sub-section 3.1, page 7, paragraph three.

Academic Editor's comment 4: Additionally, errors in notation (e.g., Equations 3 and 4) persist, impacting clarity. We encourage you to carefully address these critical issues before considering resubmission.

Authors' reply to comment 4: We appreciate the Academic Editor's insightful comment. To enhance the overall flow and clarity of the manuscript, we have addressed the comment as suggested. Kindly refer to Section 3, page 6, Equations (3) and (4).

Reviewer's comment and suggestion (1)

Comment 1: The authors have satisfactorily completed the revisions based on my questions and comments. The authors have addressed all the previous comments and concerns. Therefore, I agree with the publication of the revised paper.

Final recommendation: Accept.

Authors' reply to comment 1: Thank you very much for reviewing our paper and sharing your positive views with us. We greatly appreciate the positive evaluation provided by the reviewer regarding our manuscript. The acknowledgment of the paper being well-composed and meeting the reviewer's standards is indeed motivating.

Reviewer's comment and suggestion (2)

Note that changes made are shown in red in the revised manuscript.

Thank you very much for providing us with a constructive comment. We have addressed the comment as follows:

Comment 1: Firstly, while you acknowledge that the Rayleigh channel model does not fully capture the complexities of the THz range, there has been no substantial change in the mathematical modeling since the original submission. The Rayleigh model remains inadequate for the type of scenarios being discussed. Additionally, other phenomena affecting THz

communications are mentioned in the text, but they are not reflected in the mathematical model or the numerical results.

Authors' reply to comment 1: We agree with the Reviewer's comment. In an effort to improve the overall flow, we tried to clear the comment. Kindly see sub-section 3.1, page 7. as referred by [Reference 28]

Comment 2: Furthermore, the issue I raised in the initial review regarding the BER curves persists. As noted previously, the BER curves show exponential decay for all detectors, which does not align with the real-world behavior. In detectors like ZF or MMSE, the BER curves exhibit linear decay as the SNR increases. The results presented in the current version still contradict well-established findings in the literature.

Authors' reply to comment 2: We appreciate the Reviewer's insightful comment. To enhance the overall flow and clarity of the manuscript, we have addressed the comment as suggested. Kindly refer to Section 7, starting from page 18-20, for the revised Figure and description of Fig 7,8 and 9.

Author action: We re-evaluated the system model and simulation parameters to ensure the accuracy of the BER calculations by refining the channel model and adjusting SNR range to align with those used in comparable literature. The implementation of ZF and MMSE algorithms was verified to ensure compliance with theoretical models. Additional simulations were conducted to observe BER behavior over an extended SNR range. The revised BER results were cross-validated against established references, confirming the correction of previously observed exponential behavior to the expected linear decay. These updates were incorporated into the manuscript by revising Figures 7, 8, and 9 to reflect the corrected BER curves, enhancing the discussion in Section 7 to detail the methodological adjustments and their alignment with the literature, and emphasizing the significance of achieving the expected decay behavior for the accuracy of the presented results.

Comment 3: There are still errors in the notation (specifically in Equations 3 and 4), which further complicate the clarity of the work.

Authors' reply to comment 3: We appreciate the Reviewer's insightful comment. To enhance the overall flow and clarity of the manuscript, we have addressed the comment as suggested. Kindly refer to Section 3, page 6, Equations (3) and (4).

Reviewer's comment and suggestion (3)

Note that changes made are shown in red in the revised manuscript.

Thank you very much for providing us with a constructive comment. We have addressed the comment as follows:

Comment 1(a): Equation (2): the index appears as the upper limit. Change the upper limit.

Authors' reply to comment 1(a): We appreciate the reviewer's insightful comment. To enhance the overall flow and clarity of the manuscript, we have addressed the comment as suggested. Kindly refer to Section 3, page 6, Equations 2. Changed the upper limit from k (the index) to K (the total number of users).

Comment 1(b): Equation (3): Why isn't frequency offset considered [1-4]

Authors' reply to comment 1(b): We appreciate the reviewer's insightful comment. To enhance the overall flow and clarity of the manuscript, we have addressed the comment as suggested. Kindly refer to Section 3, page 6, Equations (3).

Comment 1(c): Try to use boldface for vectors and matrices.

Authors' reply to comment 1(c): We agree with the reviewer's comment. To enhance clarity and improve the manuscript's overall flow, we have carefully reviewed and resolved issues related to boldface for vectors and matrices throughout the paper.

Comment 1(d): Between equations (2) and (3): "v_{B_k}" and "V" are not defined. Define all variables used in all equations.

Authors' reply to comment 1(d): We appreciate the reviewer's insightful comment. To enhance the overall flow and clarity of the manuscript, we have addressed the comment as suggested. Kindly refer to Section 3, page 6, Between equations (2) and (3).

Comment 1(e): Figs 3-6: can the spectral efficiency be improved using single user massive MIMO (SU-MMIMO) [4-7]

Authors' reply to comment 1(e): We appreciate the reviewer's insightful comment. To enhance the overall flow and clarity of the manuscript, we have addressed the comment as suggested. Kindly refer to Section 7, page 17, below figure 6. as referred by [Reference 49]

Authors Action: As recommended by Reviewer 3, We have utilized this paper:

1. K. Vasudevan, K. Madhu, Shivani Singh, "Data Detection in Single User Massive MIMO Using Re-Transmissions", The Open Signal Processing Journal, vol. 6, pp. 15–26, March 2019

Comment 1(f): Figs 7-9: can the BER be improved using a turbo code [1-7]

Authors' reply to comment 1(f): We appreciate the reviewer's insightful comment. To enhance the overall flow and clarity of the manuscript, we have addressed the comment as suggested. Kindly refer to Section 7, page 20, below figure 9. as referred by [Reference 50]

Authors Action: As recommended by Reviewer 3, We have utilized this paper:

2. K. Vasudevan, "Near Capacity Signaling over Fading Channels using Coherent Turbo Coded OFDM and Massive MIMO", International Journal On Advances in Telecommunications, issn 1942-2601, vol. 10, no. 1 & 2, year 2017, 22:37

Comment 1(g): Clearly specify the channel used. Is it time-invariant or fading [8]

Authors' reply to comment 1(g): We appreciate the reviewer's insightful comment. To enhance the overall flow and clarity of the manuscript, we have addressed the comment as suggested. Kindly refer to sub-section 3.1, pages 6, paragraph one.
