# Supplementary material for: Spectral efficiency and BER analysis of RNN based hybrid precoding for cell free massive MIMO under terahertz communication
Source: PLoS One. 2025 Aug 14;20(8):e0328499. doi: 10.1371/journal.pone.0328499 (PMC12352664; doi:10.1371/journal.pone.0328499)
Supplement: Attachment — Submitted filename: PONER2.pdf [file pone.0328499.s005.pdf]

This reviewer had almost all concerns clarfied. However, one point needs further clarification.

Question 1 First we start our analysis from Figure 5, which presents curves of the system metric as a function of the total number of UEs (User Equipment) K.

The authors defined the SNR (Signal-to-Interference-plus-Noise Ratio) as

$$SINR_k = \frac{\frac{p}{k} \left|\boldsymbol{h}_{e_k} \boldsymbol{\nu}_{\boldsymbol{B}_k}\right|^2}{\sum_{k \neq j}^{K} \left|\boldsymbol{h}_{e_k} \boldsymbol{\nu}_{\boldsymbol{B}_j}\right|^2 + \sigma^2}, \tag{1}$$

then, defined the ibserved UE AR (Achievable Rate) as

$$r_k = \log_2 \left(1 + SINR_k\right). \tag{2}$$

Finally, the SE (Spetral Efficiency) of the k-th UE as

$$SE_k = \frac{r_k}{B}. \quad \text{(Verify if } B \text{ was defined before.)} \tag{3}$$

From Expression 1, one can expect that, increasing the number of UEs K, lower is the SNR fot the k-th UE. Consequently, lower is the AR and lower is the SE, which is a typical behavoir of CF (Cell-Free) systems.

Based on the aforementioned, what could explain the increasing curves presented in Figure 5?

If one defines the average UE SE by

$$\bar{SE} = \frac{1}{K} \sum_{k=1}^{K} SE_k \tag{4}$$

and the total SE by

$$SE = \sum_{k=1}^{K} SE_k, \tag{5}$$

the results presented in Figures 3∼6 are based on the SE of the k-th UE, given in Expression 3, the average SE, given in Expression 4, or the total SE, given in Expression 5?

In some situations, although the SE of the k-th UE, or the average SE of the UEs, decrease as more UEs appear in the scenario, the total SE may increase.
