# Supplementary material for: Spectral efficiency and BER analysis of RNN based hybrid precoding for cell free massive MIMO under terahertz communication
Source: PLoS One. 2025 Aug 14;20(8):e0328499. doi: 10.1371/journal.pone.0328499 (PMC12352664; doi:10.1371/journal.pone.0328499)
Supplement: Attachment — Submitted filename: Response to Reviewers.docx [file pone.0328499.s006.docx]

**Original Article Title: “Spectral Efficiency and BER Analysis of RNN based Hybrid Precoding for Cell Free Massive MIMO Under Terahertz Communication”**

**To:** PLOS ONE Editor

**Re:** Response to reviewers

Dear Editor,

Thank you for giving us the opportunity to revise the manuscript and address the reviewers’ comments.

We are uploading (a) our point-by-point response to the comments (below) (response to reviewers, under “Response to Reviewers*”*), (b) an updated manuscript with red highlighting indicating changes (as “Revised Manuscript with Track Changes*”*), and (c) a clean updated manuscript without highlights (“Manuscript”*).*

Best regards,

<Tadele A. Abose> et al.

**Academic Editor Concern # 1: When submitting your revision, we need you to address these additional requirements**

**Please ensure that your manuscript meets PLOS ONE's style requirements, including those for file naming. The PLOS ONE style templates can be found at**

<https://journals.plos.org/plosone/s/file?id=wjVg/PLOSOne_formatting_sample_main_body.pdf> and

<https://journals.plos.org/plosone/s/file?id=ba62/PLOSOne_formatting_sample_title_authors_affiliations.pdf>

**Author response:** We thank the Academic Editor for the reminder regarding PLOS ONE's style and formatting requirements. We have carefully revised the manuscript to align with PLOS ONE's formatting guidelines, including file naming conventions.

**Author action:** We have formatted the manuscript based on PLOS ONE’s style templates and instructions, ensuring that both the main document and the file naming meet the journal's requirements.

**Academic Editor Concern 2:** **Please update your submission to use the PLOS LaTeX template. The template and more information on our requirements for LaTeX submissions can be found at**

<http://journals.plos.org/plosone/s/latex>

**Author response:** We sincerely thank the Academic Editor for the careful evaluation of our manuscript. Regarding the request to update the submission to the PLOS LaTeX template, we would like to clarify that our original submission was prepared using Microsoft Word format. As per the PLOS ONE submission guidelines, MS Word is an accepted format, and we have continued our revisions in MS Word accordingly. We respectfully request permission to proceed with the submission of the revised paper in MS Word format. Thank you again for your consideration and support.

**Author action:** We have carefully revised and finalized the manuscript using Microsoft Word format, in line with the PLOS ONE submission guidelines. We have ensured that all requested edits and formatting corrections have been incorporated while continuing in the accepted MS Word format.

**Academic Editor Concern 3: Please note that PLOS ONE has specific guidelines on code sharing for submissions in which author-generated code underpins the findings in the manuscript. In these cases, we expect all author-generated code to be made available without restrictions upon publication of the work. Please review our guidelines at** <https://journals.plos.org/plosone/s/materials-and-software-sharing#loc-sharing-code> **and ensure that your code is shared in a way that follows best practice and facilitates reproducibility and reuse.**

**Author response:** We thank the Academic Editor for this important reminder regarding the code-sharing policy. We fully understand the importance of transparency, reproducibility, and reuse of research outputs.

**Author action:** We have prepared and shared the complete set of author-generated code and data set used related to our manuscript in a ZIP file format, following the PLOS ONE guidelines on code sharing.

**Academic Editor Concern 4:** **We note that the grant information you provided in the ‘Funding Information’ and ‘Financial Disclosure’ sections do not match. When you resubmit, please ensure that you provide the correct grant numbers for the awards you received for your study in the ‘Funding Information’ section.**

**Author response:** We appreciate the Academic Editor's observation. We confirm that no external funding or grant support was received for this study.

**Author action:** We have updated the 'Funding Information' and 'Financial Disclosure' sections to clearly and consistently state that no funding or grants were received**.**

**Academic Editor Concern 5: We note that your Data Availability Statement is currently as follows: All relevant data are within the manuscript and its Supporting Information files.**

Please confirm at this time whether or not your submission contains all raw data required to replicate the results of your study. Authors must share the “minimal data set” for their submission. PLOS defines the minimal data set to consist of the data required to replicate all study findings reported in the article, as well as related metadata and methods (<https://journals.plos.org/plosone/s/data-availability#loc-minimal-data-set-definition>).

For example, authors should submit the following data:

- The values behind the means, standard deviations and other measures reported;
- The values used to build graphs;
- The points extracted from images for analysis.

Authors do not need to submit their entire data set if only a portion of the data was used in the reported study.

If your submission does not contain these data, please either upload them as Supporting Information files or deposit them to a stable, public repository and provide us with the relevant URLs, DOIs, or accession numbers. For a list of recommended repositories, please see <https://journals.plos.org/plosone/s/recommended-repositories>.

If there are ethical or legal restrictions on sharing a de-identified data set, please explain them in detail (e.g., data contain potentially sensitive information, data are owned by a third-party organization, etc.) and who has imposed them (e.g., an ethics committee). Please also provide contact information for a data access committee, ethics committee, or other institutional body to which data requests may be sent. If data are owned by a third party, please indicate how others may request data access.

**Author response:** We appreciate the Academic Editor’s guidance on the data availability requirements. We confirm that all raw data necessary to replicate the results of our study have been provided.

**Author action:** We have uploaded all relevant data, including the raw values used to generate figures and tables, in a ZIP file as Supporting Information. This ensures full compliance with PLOS ONE’s minimal data set requirements.

**Reviewer 1, Concern 1: The abstract is unclear and requires significant editing. The highlights are too long in each sentence. Please shorten it in one line.**

**Author response:** We sincerely thank the reviewer for the insightful comment. We carefully revised the abstract to improve its clarity and conciseness. Each highlight has been shortened to one line as suggested.

**Author action:** We have edited the abstract and shortened the highlights to one line each.

**Reviewer 1, Concern 2: Updating references up to 2024 is required for this paper.**

**Author response:** We appreciate the reviewer’s valuable suggestion. We have updated the references to include more recent and relevant works published up to 2024.

**Author action:** We have revised and updated several references, including references [3], [7], [11], and [23], to reflect the latest developments in the field as of 2024.

**Reviewer 1, Concern 3: Improving the English language is very necessary, along with correcting grammar, proofreading, and sentence structure.**

**Author response:** We thank the reviewer for highlighting this important point. We have carefully proofread the manuscript and improved the English language, grammar, and sentence structure throughout the paper.

**Author action:** The manuscript has been thoroughly proofread by a third party with expertise in academic writing to ensure clarity, grammatical correctness, and improved sentence structure.

**Reviewer 1, Concern 4: The interpretation not sufficient in technical discussion and validation and application.**

**Author response:** We appreciate the reviewer’s valuable feedback. In response, we have expanded the technical discussion, validation, and practical application aspects in the revised manuscript. Specifically, we have strengthened the interpretation of the simulation results, linked them more explicitly to the theoretical models, and emphasized the real-world implications of the proposed method.

**Author action:** We have made significant revisions to the Results and Discussion section (pages 14 and 20) and the Conclusion section to enhance the technical depth, validation clarity, and practical application discussion.

**Reviewer 1, Concern 5: The manuscript must describe a technically sound piece of scientific research with data that supports the conclusions. Experiments must have been conducted rigorously, with appropriate controls, replication, and sample sizes. The conclusions must be drawn appropriately based on the data presented.**

**Author response:** We sincerely thank the reviewer for emphasizing the importance of scientific rigor. We confirm that the simulation experiments were conducted carefully and systematically. Multiple independent simulation runs were performed to ensure the reliability and consistency of the results. Appropriate averaging across random channel realizations and different network deployments was applied to minimize randomness and variability. The conclusions have been appropriately drawn based on the data obtained from these validated simulations.

**Author action:** We have updated the manuscript to clarify the experimental methodology, including descriptions of replication procedures, sample size considerations, and statistical averaging techniques used. This information has been added at the beginning of the "Results and Discussion" section to reinforce the scientific rigor of our work.

**Reviewer 1, Concern 6: The references are inadequate and not strong enough. They need to be revised and improved for relevance.**

<https://doi.org/10.1109/ACCESS.2019.2924531>
<https://doi.org/10.1007/s12596-024-01908-9>
<https://doi.org/10.1007/s11082-024-06692-1>

**Author response:** We thank the reviewer for the valuable suggestion regarding the references. To strengthen the quality and relevance of our citations, we have added more recent and impactful references as recommended.

**Author action:** We have cited the following references to improve the strength and relevance of the paper:

Reference [26]: <https://doi.org/10.1007/s12596-024-01908-9>

Reference [27]: <https://doi.org/10.1007/s11082-024-06692-1>

Additionally, we have carefully reviewed and updated other references to enhance the overall quality of the manuscript.

**Reviewer 1, Concern 7: There are areas that require further practical discussion and better linking of the results discussion together.**

**Author response:** We thank the reviewer for this insightful comment. In response, we have carefully revised the Results and Discussion section to ensure better coherence between simulation setups, theoretical expectations, and observed outcomes. Specifically, we strengthened the practical interpretation of the simulation figures by explicitly linking them to the system model, precoding algorithms, and machine learning training phases. Furthermore, we clarified how improvements in spectral efficiency and BER are achieved through the proposed RNN-based hybrid precoding method in various realistic scenarios.

**Author action:** We revised the Results and Discussion section to emphasize the role of the extensive machine learning dataset, better link theoretical models with simulation trends (Figures 3–9), and highlight practical insights, including computational complexity and real-world deployment implications.

**Reviewer 2, Concern 1: Introduction, paragraph eight:**

**"The main contributions of this paper are, this paper introduces..."**

**Why not just start with?**

**"This paper introduces..."**

**as the paragraph below already stats:**

**"The main contributions of the paper are summarized..."**

**Author response:** We appreciate the reviewer’s careful observation. We agree with the suggestion and have modified the sentence to improve clarity and avoid redundancy.

**Author action:** We have revised the beginning of paragraph 8 in the Introduction section (Page 2). The sentence now directly starts with "This paper introduces..." as recommended.

**Reviewer 2, Concern 2: Figure 1:**

**Figure one may be misreading. Are both side different clusters of UEs and APs (if so the author better to change the APs and UEs labels for each side)? Or are both side different system configurations (if so, indicate it)?**

**Author response:** We thank the reviewer for the thoughtful comment. To clarify, both sides of Figure 1 represent different system configurations while using the same set of UEs and APs. The labels are intentionally kept identical to emphasize that the comparison is based on the network architecture rather than on user or access point identity.

**Author action:** We have updated the figure caption to explicitly state that both sides illustrate different system configurations with the same UEs and APs, and made the explanation clearer in the manuscript text.

**Reviewer 2, Concern 3: Are the terms Mobile Station (MS) and User Equipment (UE) referred to the same entity? If so, uniformize and adopt one instead of mixing UE and MS throughout the text.**

**Author response:** We thank the reviewer for the helpful observation. We confirm that Mobile Station (MS) and User Equipment (UE) refer to the same entity. To avoid confusion, we have standardized the terminology across the paper.

**Author action:** We have carefully revised the manuscript to consistently use the term "User Equipment (UE)" throughout the text, replacing all instances of "Mobile Station (MS)".

**Reviewer 2, Concern 4: Section: 3.2 Problem Formulation:**

**In the paragraph one, remove the single parenthesis after mMIMO**

**Author response:** We appreciate the reviewer’s careful attention to detail. We have removed the unnecessary single parenthesis after "mMIMO" as suggested.

**Author action**: The correction has been made in Section 3.2, paragraph one, on page 8 of the manuscript.

**Reviewer 2, Concern 5: Define** $\boldsymbol{(\cdot)}^{\boldsymbol{H}}$**,** $\boldsymbol{(\cdot)}^{\boldsymbol{T}}$ **and CN(0,1)**

**Author response:** We thank the reviewer for pointing out the need for clarification. We have added definitions for the notations as requested.

**Author action:** The notation ${(\cdot)}^{H}$ and ${(\cdot)}^{T}$ has been defined in Section 3, page 6, and *CN(0,1)* has been defined in Section 7 of the manuscript.

**Reviewer 2, Concern 6: Channel Model 3rd paragraph:**

**"The data set used for training includes real and imaginary channel gains, channel phase, spatial correlation, phase shift, correlation" - Is the second "correlation" a time related correlation?**

**Author response:** We appreciate the reviewer’s question. To clarify, the second mention of "correlation" refers to spatial correlation between antenna elements, not time-domain correlation. Time-related correlation was not explicitly modeled in this dataset.

**Author action:** We have revised the sentence for clarity to specify that the second "correlation" refers to spatial correlation between antenna elements, and we have clarified that time-related correlation was not modeled.

**Reviewer 2, Concern 7: The authors states: "Each AP server’s K UEs".**

**Does it mean that, if more than K UEs (let's say S) are placed in the scenario, an AP selection technique must be applied to assign K UEs, out of S, to each AP, or does it mean that the author modeled the system with a limited number of K UEs? Assigning a sub set of UEs to each AP, referred to as User-Centric Cell-Free, is known for providing a system scenario with reduced interference.**

**Author response:** We appreciate the reviewer’s insightful comment. The statement "Each AP serves K UEs" refers to a system model where the total number of UEs assigned to each AP is fixed to K. We modeled the system with a limited number of K UEs per AP for simplicity and scalability in the simulation. No AP selection or dynamic user association was applied. If more than K UEs were placed, an AP selection technique could indeed be applied, but it was not part of this model.

**Author action:** We have clarified the statement and provided additional context in Section 3, page 5, to indicate that the number of UEs per AP is fixed for the simplicity of our model.

**Reviewer 2, Concern 8: The authors name the entity described in Eq. (7) as sum rate. However, that entity is the achievable rate of the *kth* UE instead. The authors must be careful with such definitions because while explaining Figure 5, for example, the authors state that the Spectral Efficiency (SE) (which, in this case, was derived from the achievable rate) increases with the number of UEs. However, as can be inferred from Eq. (6), the SINR decreases by increasing K and this leads to a lower Achievable rate (consequently a lower SE). However, the sum rates indeed increase and presents a saturation trend. So, are the results presented in figures 1~5 indeed SE or are they sum rates?**

**Author response:** We appreciate the reviewer’s valuable feedback. You are correct that the entity described in Eq. (7) should be referred to as the achievable rate of the *Kth* UE rather than the sum rate. Additionally, we acknowledge the discrepancy between spectral efficiency (SE) and sum rate in the context of Figures 1-5.

**Author action:** We have revised Equation (6) to correctly define the achievable data rate, and we have changed the references to spectral efficiency in Figures 1-5 to indicate the sum rate instead.

**Reviewer 2, Concern 9:** **Although the authors states Terahertz (THz) communications are characterized by Line-of-Sight (LoS) components, in Eq (20) and in the first paragraph of the Section 7 (Results and Discussion) the channel gain is characterized by a complex normal distribution with zero mean, CN (0,1), which is a Rayleigh distribution. A proper characterization must be applied to enrich the simulated analysis.**

**Author response:** We thank the reviewer for raising this important issue. As stated, THz communications are typically characterized by a Line-of-Sight (LoS) component. The reference to CN (0,1) in Eq. (20) and Section 7 was meant to simplify the small-scale fading component in certain analytical contexts, not to represent the full channel model used in our simulations.

**Author action:** We have clarified in Section 3.1 (page 7) that a geometric channel model with scatterers is used for THz communications, which explicitly incorporates LoS and sparse multipath effects. This model is fundamentally different from the Rayleigh fading model and better represents the THz channel characteristics.

**Reviewer 2, Concern 10: In Eq. (10) the system optimization was considered to maximize the UEs sum rate, what does not focus in the UE quality of service equalization. This means that, either the scenario present UEs in a very good channel condition or a poor channel condition. Based in this statement, the extracted metrics must be analyzed carefully. For example, how were the Bit Error Rate (BER) curves generated? Are those curves extracted from the same UE selected in the scenario just varying the transmitted power? Are those curves the average value of the BERs of all the UEs?**

**Author response:** We appreciate the reviewer’s valuable feedback. The BER curves shown in the manuscript represent the average BER across all UEs, computed under varying transmit power conditions while maintaining the same scenario configuration. As noted, this system optimization does not incorporate per-user quality-of-service (QoS) equalization, which may lead to better performance for users in favorable channel conditions.

**Author action:** We have clarified this point below Eq. (10) on page 8, stating that this design choice focuses on spectral efficiency improvements and that fairness-aware formulations will be considered in future work. the BER curves reflect the average BER for all UEs. this clarification is repeated on page 18.
