# Supplementary material for: Spectral efficiency and BER analysis of RNN based hybrid precoding for cell free massive MIMO under terahertz communication
Source: PLoS One. 2025 Aug 14;20(8):e0328499. doi: 10.1371/journal.pone.0328499 (PMC12352664; doi:10.1371/journal.pone.0328499)
Supplement: Attachment — Submitted filename: Response_to_Reviewers_auresp_2.docx [file pone.0328499.s007.docx]

**Original Article Title: “Spectral Efficiency and BER Analysis of RNN based Hybrid Precoding for Cell Free Massive MIMO Under Terahertz Communication”**

**To:** PLOS ONE Editor

**Re:** Response to reviewers

Dear Editor,

Thank you for giving us the opportunity to revise the manuscript and address the reviewers’ comments.

We are uploading (a) our point-by-point response to the comments (below) (response to reviewers, under “Response to Reviewers*”*), (b) an updated manuscript with red highlighting indicating changes (as “Revised Manuscript with Track Changes*”*), and (c) a clean updated manuscript without highlights (“Manuscript”*).*

Best regards,

<Tadele A. Abose> et al.

**Reviewer Concern 1:** $\boldsymbol{SE}_{\boldsymbol{k}}\boldsymbol{=}\frac{\boldsymbol{r}_{\boldsymbol{k}}}{\boldsymbol{B}}$**(Verify if B was defined before)**

**Author response:** We appreciate the reviewer’s attention to detail. The variable **B** in Equation (3) indeed denotes the system bandwidth. Although it is a standard notation, we acknowledge that it was not explicitly defined upon first use. We have now updated the manuscript to clearly define **B** as the system bandwidth to improve clarity.

**Author action:** We revised the text immediately following Equation (3) as in page 6.

**Reviewer Concern 2: From Expression (1), one can expect that increasing the number of UEs K lowers SINR, and thus reduces SE. However, in Figure 5, SE appears to increase with K. Which SE metric is plotted:** $\boldsymbol{SE}_{\boldsymbol{k}}$**, average SE, or total SE?**

**Author response:** We thank the reviewer for the thoughtful observation regarding the trend in Figure 5. The curve in Figure 5 reflects the total spectral efficiency (SE**)**, computed as:

$\boldsymbol{SE}_{\boldsymbol{k}}\boldsymbol{=}\frac{\boldsymbol{r}_{\boldsymbol{k}}}{\boldsymbol{B}}$

As more users (UEs) are added to the system, while the individual or average spectral efficiency may decrease due to increased interference and resource contention, the total SE can increase since it sums the contributions from all users. This cumulative gain is consistent with CFMM systems, which scale network throughput as user density grows. We agree that explicitly stating this is necessary for clarity.

**Author action:** We revised the manuscript in the Results and Discussion section under Figure 5 on page 16.
